# Criteria for identifying residual tumours after neoadjuvant chemotherapy of breast cancers: a magnetic resonance imaging study

**DOI:** 10.1038/s41598-020-79743-8

**Published:** 2021-01-12

**Authors:** Yunju Kim, Sung Hoon Sim, Boram Park, In Hye Chae, Jai Hong Han, So-Youn Jung, Seeyoun Lee, Youngmi Kwon, In Hae Park, Kyounglan Ko, Chan Wha Lee, Keun Seok Lee, Han-Sung Kang, Eun Sook Lee

**Affiliations:** 1 Department of Radiology, CHA Bundang Medical Center, CHA University, Seongnam, Korea; 2 Division of Radiology, Center for Breast Cancer, National Cancer Center, Goyang, Korea; 3 Division of Hemato-Oncology, Center for Breast Cancer, National Cancer Center, 323 Ilsan-ro, Ilsandong-gu, Goyang-si, 10408 Gyeonggi-do Korea; 4 Division of Translational Science, Research Institute, National Cancer Center, Goyang, Korea; 5 Biostatistics Collaboration Team, Research Core Center, Research Institute, National Cancer Center, Goyang, Korea; 6 Division of Cancer Epidemiology and Management, Research Institute, National Cancer Center, Goyang, Korea; 7 Statistics and Data Center, Samsung Medical Center, Seoul, Korea; 8 Division of Surgery, Center for Breast Cancer, National Cancer Center, Goyang, Korea; 9 Division of Pathology, Center for Breast Cancer, National Cancer Center, Goyang, Korea; 10 Division of Internal Medicine, Department of Hemato-Oncology, Korea University College of Medicine, Seoul, Korea

**Keywords:** Cancer, Breast cancer

## Abstract

We investigated magnetic resonance imaging (MRI) criteria identifying residual tumours in patients with triple-negative and human epidermal growth factor receptor type 2-positive (HER2+) breast cancer following neoadjuvant chemotherapy. Retrospectively, 290 patients were included who had undergone neoadjuvant chemotherapy and definitive surgery. Clinicopathological features, as well as lesion size and lesion-to-background parenchymal signal enhancement ratio (SER) in early- and late-phase MRIs, were analysed. Receiver operating characteristic (ROC) analyses evaluated diagnostic performances. Maximal MRI values showing over 90% sensitivity and negative predictive value (NPV) were set as cut-off points. Identified MRI criteria were prospectively applied to 13 patients with hormone receptor-negative (HR-) tumours. The lesion size in HR-HER2-tumours had the highest area under the ROC curve value (0.92), whereas this parameter in HR + HER2 + tumours was generally low (≤ 0.75). For HR-tumours, both sensitivity and NPV exceeded the 90% threshold for early size > 0.2 cm (HR-HER2-) or > 0.1 cm (HR-HER2 +), late size > 0.4 cm, and early SER > 1.3. In the prospective pilot cohort, the criteria size and early SER did not find false negative cases, but one case was false negative with late SER. Distinguishing residual tumours based on MRI is feasible in selected triple-negative and HER2 + breast cancer patients.

## Introduction

Neoadjuvant chemotherapy (NAC) is widely used in breast cancer patients because it reduces tumour burden prior to definitive surgery, and survival outcomes improve in patients with pathological complete response (pCR)^1–3^. With the introduction of various target and cytotoxic agents into clinical practice, pCR rates have increased, especially in human epidermal growth factor receptor type 2-positive (HER2+) and triple-negative (TN) but not in hormone receptor-positive (HR+) breast cancers^4^.

Imaging techniques are useful for evaluating the response to neoadjuvant treatment. Among them, magnetic resonance imaging (MRI) is known as the most accurate imaging modality for the response assessment of breast cancer^5–7^. As of now, however, the overall ability of MRI is not sufficient for an accurate pCR prediction^5,8^. But there have been several attempts to determine its feasibility for the identification of exceptional responders^9,10^. Several studies have reported that the diagnostic performance of MRI to identify a pCR is high in TN and HER2+ breast cancers, whereas it is low in HR+ tumours^5,11–13^.

The size and lesion-to-background parenchymal signal enhancement ratio (SER) have been suggested to assess the residual tumour and pCR^14,15^. If imaging modalities could reliably identify a pCR after NAC, omitting surgery may become feasible. Besides, performing additional presurgical needle biopsies in radiological complete response (rCR) cases after NAC before surgery may improve the diagnostic accuracy^16^. The purpose of this study was to investigate MRI assessment criteria for detecting residual tumours in patients with TN and HER2+ breast cancer after NAC and to apply these preliminary criteria to a pilot cohort.

## Results

### Patient characteristics and histopathologic results

The clinical and pathologic results are summarized in Table 1. Fifty percent of the patients (145 of 290 patients) achieved a breast pCR: no residual tumour in 107 patients and residual ductal carcinoma in situ (DCIS) in 38 patients. Before treatment, the majority of the study population had invasive ductal carcinoma (284 of 290). The pCR rates differed depending on the histologic grade, HR status, and subtypes. Higher-grade and HR- tumours showed better treatment responses. Among the subtypes, HR-HER2 + tumours achieved the highest pCR rate (62 of 90 patients), whereas HR + HER2 + tumours showed the lowest pCR rate (42 of 103 patients). The most frequently used NAC regimens were anthracycline-taxane combinations (174 of 290). Of 193 patients with HER2 + cancer, 190 patients received an anti-HER2 agent (trastuzumab with/without pertuzumab) in addition to the chemotherapy regimen. Seventy-nine percent of patients (228 of 290) underwent breast-conserving surgery.

**Table 1:** Clinicopathologic findings according to the pathological response.

Characteristic	Total (n = 290)	pCR (n = 145)	non-pCR (n = 145)	p-value
Age (y, mean ± standard deviation)	50.5 ± 9.5	50.9 ± 9.5	50.1 ± 9.5	0.4664
**Clinical TNM stage**				0.0188
II	150	85 (56.7)	65 (43.3)	
III	140	60 (42.9)	80 (57.1)	
**Clinical T stage**				0.1566
1	8	4 (50.0)	4 (50.0)	
2	208	112 (53.9)	96 (46.2)	
3	61	25 (41.0)	36 (59.0)	
4	13	4 (30.8)	9 (69.2)	
**Histologic type**				0.6842
Invasive ductal carcinoma	284	143 (50.4)	141 (49.7)	
Others	6	2 (33.3)	4 (66.7)	
**Histologic grade**				0.0103
1	1	1 (100.0)	0 (0.0)	
2	109	43 (39.5)	66 (60.6)	
3	175	97 (55.4)	78 (44.6)	
Unknown	5	4 (80.0)	1 (20.0)	
**HR (ER and/or PgR)**				0.0197
Negative	187	103 (55.1)	84 (44.9)	
Positive	103	42 (40.8)	61 (59.2)	
**HER2**				0.0619
Negative	97	41 (42.3)	56 (57.7)	
Positive	193	104 (53.9)	89 (46.1)	
**Subtype**				< 0.0001
HR-HER2−	97	41 (42.3)	56 (57.7)	
HR-HER2 +	90	62 (68.9)	28 (31.1)	
HR + HER2 +	103	42 (40.8)	61 (59.2)	
**Chemotherapy regimen**				0.0109
Anthracycline + taxane	174	84 (48.3)	90 (51.7)	
Anthracycline + taxane + platinum	45	16 (35.6)	29 (64.4)	
Taxane + platinum	71	45 (63.4)	26 (36.6)	
**Breast surgery**				0.7745
Breast-conserving surgery	228	115 (50.4)	113 (49.6)	
Total mastectomy	62	30 (48.4)	32 (51.6)	

Unless indicated otherwise, data represent the number of patients (percentage).

*pCR* pathological complete response, *HR* hormone receptor, *ER* oestrogen receptor, *PgR* progesterone receptor, *HER2* human epidermal growth factor receptor 2.

### Imaging findings and diagnostic performance

Table 2 presents the imaging findings in the post-NAC MRI. In most patients, the background parenchymal enhancement (BPE) was classified as either minimal (178 of 290) or mild (109 of 290), and there was no significant difference between pCR and non-pCR groups. In the pCR group, the median size of the enhancing lesion was 0 cm vs. 0.3 cm (early vs. late phase), and the median SER was 1.1 vs. 1.2 (early vs. late phase).

**Table 2:** MRI findings according to the pathological response.

Characteristic	Total (n = 290)	pCR (n = 145)	non-pCR (n = 145)	p-value
**BPE**				0.7193
Minimal	178	87 (48.9)	91 (51.1)	
Mild	109	57 (52.3)	52 (47.7)	
Moderate	3	1 (33.3)	2 (66.7)	
**Enhancing lesion, early phase**				< 0.0001
No (size = 0 cm)	97	83 (85.6)	14 (14.4)	
Yes (size > 0 cm)	193	62 (32.1)	131 (67.9)	
**Enhancing lesion, late phase**				< 0.0001
No (size = 0 cm)	77	69 (89.6)	8 (10.4)	
Yes (size > 0 cm)	213	76 (35.7)	137 (64.3)	
Early size (cm)	0.6 (0–9.4)	0 (0–6.0)	1.2 (0–9.4)	< 0.0001
Late size (cm)	0.8 (0–11.0)	0.3 (0–11.0)	1.3 (0–9.4)	< 0.0001
Early SER	1.8 (0.3–7.3)	1.1 (0.3–5.6)	2.3 (0.6–7.3)	< 0.0001
Late SER	1.7 (0.4–4.1)	1.2 (0.4–4.1)	2.0 (0.7–3.7)	< 0.0001

The data are expressed as the number of patients (percentage) for categorical variables and as median (range) for continuous variables.

*pCR* pathological complete response, *BPE* background parenchymal enhancement, *SER* lesion-to-background parenchymal signal enhancement ratio.

**Figure 1:**
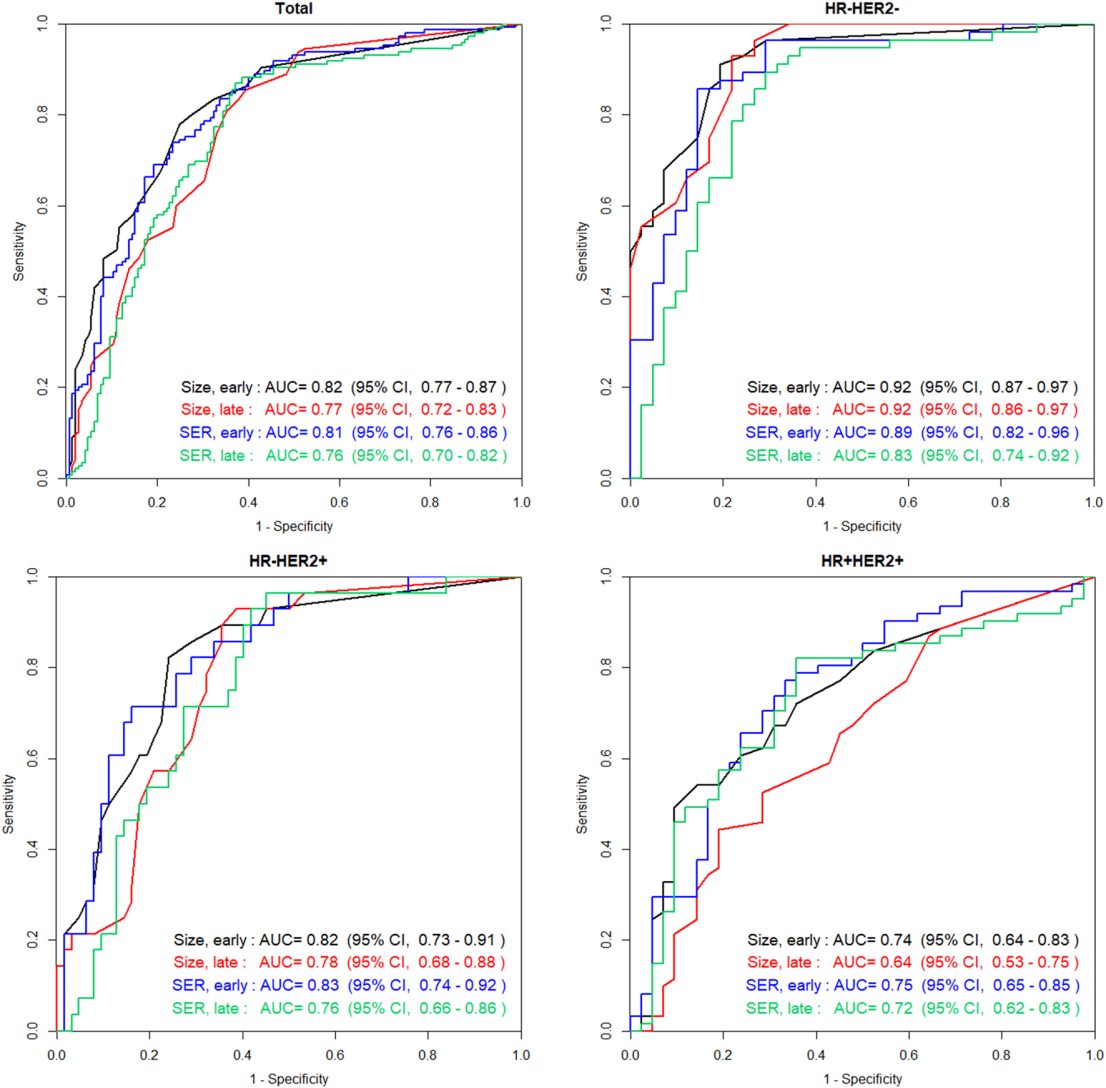
ROC curves and AUC values for identifying residual lesions. *ROC* receiver operating characteristic, *AUC* area under the curve, *HR* hormone receptor, *HER2* human epidermal growth factor receptor 2, *SER* lesion-to-background parenchymal signal enhancement ratio.

Table 3 and Fig. 1 show the area under the receiver operating characteristic curve (AUC) values for detecting a residual lesion according to size and SER in the early and late phases. When comparing size vs. SER, there was no significant difference (0.82 vs. 0.81, p = 0.6995 in the early phase; 0.78 vs. 0.76, p = 0.6102 in the late phase). In the comparison early vs. late phases, the early phase showed higher AUC values (0.82 vs. 0.78 by size, p = 0.0081; 0.81 vs. 0.76 by SER, p = 0.0018). In the analysis by subtype, the HR-HER2- lesion size had the highest AUC value (0.92). As the AUC values of HR + HER2 + tumours were generally low (≤ 0.75), this subtype was excluded from subsequent analyses that established the preliminary diagnostic criteria.

**Table 3:** Comparison of AUC values for identifying residual lesions.

	AUC		All types	p-value	HR- HER2-	p-value	HR- HER2 +	p-value	HR + HER2 +	p-value
Size vs. SER	Early phase	Size	0.82	0.6995	0.92	0.2866	0.82	0.9246	0.74	0.7781
		SER	0.81		0.89		0.83		0.75	
	Late phase	Size	0.78	0.6102	0.92	0.0305	0.78	0.7273	0.64	0.1216
		SER	0.76		0.83		0.76		0.73	
Early vs. Late phase	Size	Early phase	0.82	0.0081	0.92	0.9602	0.82	0.1799	0.74	0.0069
		Late phase	0.78		0.92		0.78		0.64	
	SER	Early phase	0.81	0.0018	0.89	0.0533	0.83	0.0373	0.75	0.4399
		Late phase	0.76		0.83		0.76		0.73	

*AUC* area under the curve, *HR* hormone receptor, *HER2* human epidermal growth factor receptor 2, *SER* lesion-to-background parenchymal signal enhancement ratio.

For HR-HER2− and HR-HER2 + subtypes, some representative cut-off points for detecting residual lesions are presented in Table 4 (see also Supplementary Table). Sensitivity and negative predictive value (NPV) were both above 90% when one of the following parameters were defined as positive MRI findings: early size > 0.2 cm (HR-HER2−) or > 0.1 cm (HR-HER2+), late size > 0.4 cm, early SER > 1.3, and late SER > 0.8 (HR-HER2−) or > 1.5 (HR-HER2+).

**Table 4:** Preliminary cut-off points for identifying residual lesions.

		Cut-off	Sensitivity	Specificity	Accuracy	PPV	NPV
HR-HER2-	Early size (cm)	0.2	94.6	73.2	85.6	82.8	90.9
	Late size (cm)	0.4	96.4	73.2	86.6	83.1	93.8
	Early SER	1.3	96.4	70.7	85.6	81.8	93.5
	Late SER	0.8	100.0	7.3	60.8	59.6	100.0
HR-HER2 +	Early size (cm)	0.1	92.9	54.8	66.7	48.1	94.4
	Late size (cm)	0.4	92.9	61.3	71.1	52.0	95.0
	Early SER	1.3	92.9	51.6	64.4	46.4	94.1
	Late SER	1.5	92.9	58.1	68.9	50.0	94.7

*PPV* positive predictive value, *NPV* negative predictive value, *HR* hormone receptor, *HER2* human epidermal growth factor receptor 2, *SER* lesion-to-background parenchymal signal enhancement ratio.

### Prospective application

**Figure 2:**
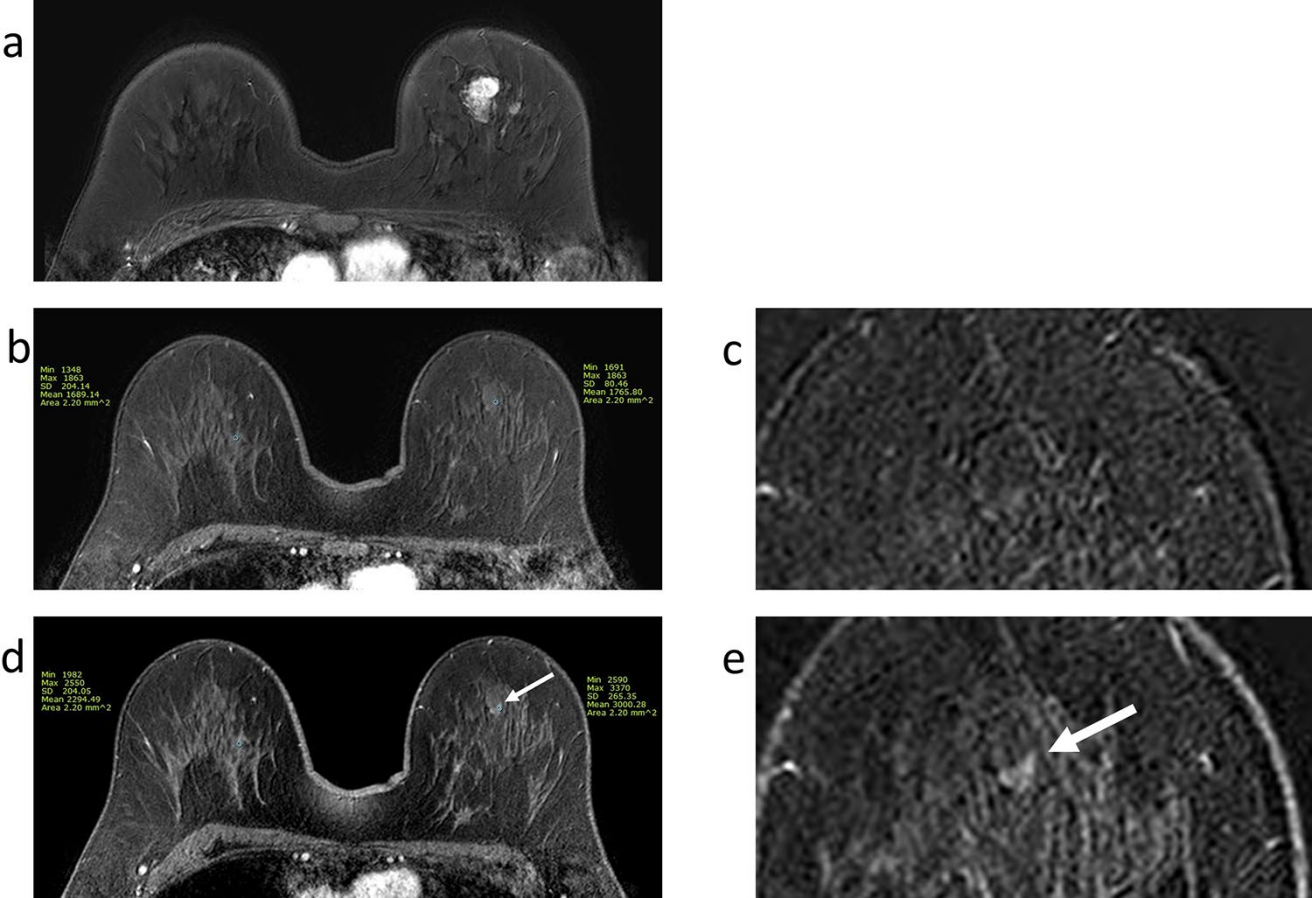
A 44-year-old woman with HR-HER + invasive ductal carcinoma. After 8 cycles of neoadjuvant chemotherapy (NAC, 4 cycles of anthracycline/cyclophosphamide followed by 4 cycles of taxane/trastuzumab), this patient received breast-conserving surgery. A 0.1-cm-sized ductal carcinoma in situ (DCIS) was found in the surgical specimen. **a** Pre-NAC magnetic resonance imaging (MRI) revealed an irregular enhancing mass in the left breast. **b** After completion of the NAC, the early post-contrast T1-weighted image showed no enhancing lesion. **d** In the late phase, a 0.7-cm-sized enhancing lesion was observed at the original tumour site (arrow). **c**,**e** are magnified subtraction images of each phase. Lesion-to-background signal enhancement ratios (SERs) were 1.1 in the early phase and 1.3 in the late phase. The presurgical core needle biopsy was diagnosed as atypical apocrine adenosis. According to our definitions of pathological complete response and the preliminary criteria, the MRI findings according to the criteria early size, early SER, and late SER were true negatives. Although the finding according to the criterion late size was a false positive, it may be suggestive of the residual DCIS.

**Figure 3:**
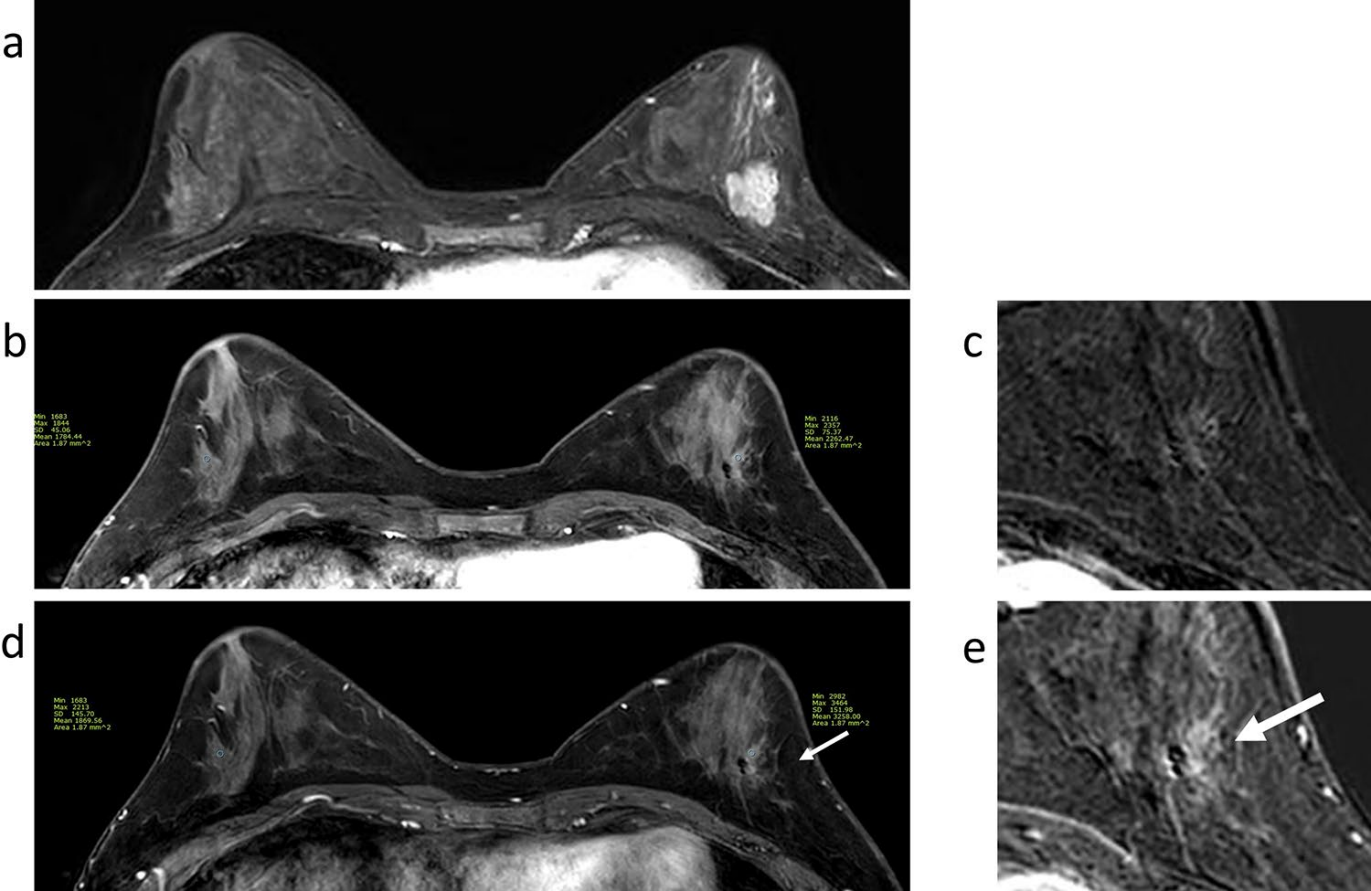
A 33-year-old woman with HR-HER− invasive ductal carcinoma. After 8 cycles of neoadjuvant chemotherapy (NAC, 4 cycles of anthracycline/cyclophosphamide followed by 4 cycles of paclitaxel/cisplatin), this patient received breast-conserving surgery. No residual tumour was found in the surgical specimen. **a** Pre-NAC magnetic resonance imaging (MRI) showed an irregular enhancing mass in the left breast. **b** After NAC completion, the early post-contrast T1-weighted image displayed no enhancing lesion around the signal void of an inserted marker clip. **d** In the late phase, a 1-cm-sized enhancing lesion was observed at the original tumour site (arrow). **c**,**e** are magnified subtraction images of each phase. Lesion-to-background signal enhancement ratios (SERs) were 1.3 in the early phase and 1.7 in the late phase. The presurgical core needle biopsy was diagnostic of a fibroadenoma. According to our definitions of pathological complete response and the preliminary criteria, the MRI findings according to the criteria early size and early SER were true negatives, whereas late size and late SER were false positives.

Table 5 shows the data of the prospective pilot cohort (see also Supplementary Figure). Of the 15 enrolled participants, nine patients achieved a breast pCR: no residual tumour in six patients and residual DCIS in three patients. As outlined in the previous section, we identified four diagnostic criteria depending on lesion size and MRI phase (Figs. 2 and 3). Different cut-off points were used for HR-HER2− (n = 4) and HR-HER2 + (n = 9) cases, whereas these criteria were not applicable for HR+ HER + tumours (n = 2). When applying the preliminary criteria, one case was false negative according to the late SER criterion (Case No. 11). However, this case showed true positive findings for size (early and late) and early SER criteria.

**Table 5:** Application of the preliminary criteria to the prospective cohort.

Case	Subtype	Early size		Late size		Early SER		Late SER		Presurgical CNB	Surgery
12	HR-HER2-	0	TN	0	TN	0.9	TN	0.9	FP	Fibrocystic change	pCR
13	HR-HER2-	0	TN	1.0	FP	1.3	TN	1.7	FP	Fibroadenoma	pCR
03	HR-HER2 +	0	TN	0	TN	0.9	TN	1.0	TN	Stromal fibrosis	pCR
06	HR-HER2 +	0	TN	0	TN	0.8	TN	0.9	TN	Fibrocystic change	pCR
09	HR-HER2 +	0	TN	0.3	TN	0.9	TN	0.9	TN	Sclerosing adenosis	pCR
14	HR-HER2 +	0	TN	0	TN	1.1	TN	1.0	TN	Stromal fibrosis	pCR
01	HR-HER2 +	0	TN	0.7	FP	1.3	TN	1.3	TN	Atypical apocrine adenosis	DCIS (0.1 cm)
02	HR-HER2 +	0.8	FP	0.8	FP	2.0	FP	2.4	FP	Fibrocystic change	DCIS (0.2 cm)
08	HR-HER2 +	0	TN	0.4	TN	1.1	TN	2.0	FP	Not performed	DCIS (0.5 cm)
11	HR-HER2 +	0.5	TP	0.5	TP	1.8	TP	1.1	FN	Not performed	microIDC
04	HR + HER2 +	0.4	NA	0.4	NA	2.0	NA	2.3	NA	DCIS	microIDC
05	HR-HER2-	4.5	TP	4.5	TP	3.6	TP	2.4	TP	Not performed	IDC (0.4 cm)
07	HR-HER2-	0.8	TP	0.8	TP	3.1	TP	2.4	TP	Not performed	IDC (0.8 cm)
10	HR-HER2 +	1.0	TP	2.0	TP	2.2	TP	1.8	TP	Not performed	IDC (0.2 cm)
15	HR + HER2 +	2.0	NA	2.0	NA	1.7	NA	1.9	NA	Not performed	IDC (1.7 cm)
TP		4		4		4		3			
TN		8		6		8		5			
FP		1		3		1		4			
FN		0		0		0		1			
NA		2		2		2		2			

Cut-off points as positive MRI findings: early size > 0.2 cm (HR-HER2-) or > 0.1 cm (HR-HER2 +), late size > 0.4 cm, early SER > 1.3, and late SER > 0.8 (HR-HER2-) or > 1.5 (HR-HER2 +).

*SER* lesion-to-background parenchymal signal enhancement ratio, *CNB* core needle biopsy, *HR* hormone receptor, *HER2* human epidermal growth factor receptor 2, *TP* true positive, *TN* true negative, *FP* false positive, *FN* false negative, *DCIS* ductal carcinoma in situ, *pCR* pathological complete response, *IDC* invasive ductal carcinoma, *NA* not applicable (unable to apply diagnostic criteria in this study).

A presurgical core needle biopsy was performed in 9 of the 15 patients. There was no residual invasive cancer in 8 patients with benign presurgical biopsy results. Among them, one case of atypical apocrine adenosis identified by presurgical biopsy was confirmed at surgery as a 0.1 cm DCIS (Case No. 01), whereas another case characterised by presurgical biopsy as DCIS was a microinvasive cancer (Case No. 04).

## Discussion

In an era where personalized care is becoming more important, there are demands to minimize treatment, but it remains challenging to reliably identify pCR before surgery. This study was initiated in view of the possibility to select appropriate candidates for post-NAC surgery omission by considering their tumour subtypes and carefully analysing their images. Our study demonstrates that simple size and SER measurements have a high ability to identify residual tumours in HR− breast cancer. In particular, the AUCs of TN lesion sizes on MRI were above 90. In HR-HER2 + tumours, size and SER on MRIs also showed AUCs of about 80.

Our study demonstrated that the lesion size in the MRI generated high AUC values. This is in agreement with the results from the American College of Radiology Imaging Network 6657 Trial^14^. They compared various preoperative measurements of the residual tumour including the longest diameter in mammography, MRI, and clinical examinations, as well as the functional volume on MRI. The study initially showed that functional tumour volume measured by MRI was strong predictor of recurrence-free survival^17^. However, they found that the longest diameter on early-phase MRI (AUC = 0.76) is more accurate for prediction of pCR than other measurements (0.69–0.70).

We did not find significant differences between the AUCs of size and SER. The definition of MRI negativity after NAC differs across studies^6^. Some studies define the complete absence of enhancement as MRI-negative, whereas others regard enhancement similar to that of normal parenchymal tissue as MRI-negative. Moreover, considering that there may also be an influence of the BPE, visual assessment can be somewhat subjective. In this respect, the parameter SER may be helpful in some ambiguous cases by providing more objective criteria.

Kim et al. reported that the SER can distinguish pCR from minimal residual cancer after NAC^15^. In their study, an early SER value of ≤ 1.6 (AUC = 0.686–0.709) shows a better diagnostic performance compared to the conventional criterion of no delayed enhancement (AUC = 0.585–0.599). They suggest that the combined criterion of SER ≤ 1.6 and size ≤ 0.2 cm could be used in a study to identify candidate patients to avoid surgery.

In our study, size and SER on early-phase images showed higher AUCs compared to those on late-phase MRIs. This is consistent with previous data by Kim et al.^13^. They measured the tumour size and found a higher AUC on early-phase MRI compared to conventional delayed-phase MRI (0.75 vs. 0.68, p = 0.002) in determining pCR. Additionally, they reported that total tumour size (both invasive and in situ components) showed higher agreement on delayed-phase than early-phase MRIs. In pretreatment MRIs of patients not receiving NAC, the tumour size is usually measured in the early phase to maximize the contrast to the normal parenchymal enhancement. In an NAC setting, however, the enhancement rate of the residual tumour might be delayed because of antiangiogenic drug effects^18,19^. Given that normal breast tissues also tend to show increased enhancement in the delayed phase, it can be assumed that results in the delayed phase are affected by a number of confounding factors.

The appropriate cut-off points of diagnostic criteria may differ according to the purpose of the study. As we intended to select patients who could safely omit surgery, we set rather strict criteria. This approach has also led to several false positive results. We presented several diagnostic criteria in this study. Due to the insufficient validation, it is difficult to conclude conclusively at present how much of the diagnostic criteria must be satisfied to safely omit surgery. However, early-phase parameters seem to be more useful compared to late-phase parameters to exclude invasive tumours.

Many studies reported differences in diagnostic performance for the evaluation of residual breast cancer according to tumour subtypes^20–22^. In a study with 746 women who underwent NAC, De Los Santos et al. found the highest NPVs in HR-HER2 + (62%) and TN (60%) tumours^20^. In a study of HER2 + breast cancer after NAC, van Ramshorst et al. showed a better NPV in HR- compared to HR + tumours (88% vs. 57%, respectively)^21^. The decreased probability of false negatives and the better NPV of HR− tumours are attributed to a higher pCR rate. Our study also demonstrated that the diagnostic MRI performance tended to be generally low in HR + HER2 + tumours. These tumours may be unsuitable candidates for predicting pCR without surgery.

This study has several limitations. One important limitation is the lack of consideration of residual DCIS and axillary lymph node metastasis. Although residual DCIS is known to have no significant impact on the overall survival rate in the neoadjuvant setting^2^, little is known about the outcome if this DCIS is left unresected. In our cohort, 5 of 145 (3.4%) cases with a breast pCR did not achieve an axillary pCR. Although axillary pCR is known to have an important influence on patient survival^2^, this study focused on the assessment of the breast lesion. Several researchers investigated imaging assessments of axillary responses^23^. Further studies are required to evaluate combined breast and axillary pCRs. Insufficient validation is another limitation of the current study. Although promising results were observed when the preliminary criteria were applied to the prospective study cohort, the sample size was too small for statistical analyses. In addition, further investigations in patients with rCR are needed on the supplementary gain of additional presurgical needle biopsies. Our study included cases where it was difficult to select the precise location for the presurgical needle biopsy, depending on the morphologic feature, extent, or multiplicity of the lesion, even though the biopsy was performed by experienced radiologists. Apart from the MRI accuracy, the implementation of a presurgical needle biopsy could be limited if the lesion is a non-mass type, extensive, or multifocal. In our study, the supplementary role of presurgical needle biopsies in diagnosing residual invasive cancers did not seem to be particularly large. Further studies are required on the feasibility and utility of presurgical needle biopsies.

In conclusion, we found that lesion size and SER on MRI identified residual tumours with high sensitivity and NPV in HR- breast cancer patients treated with NAC. These results suggest the feasibility of this approach to select appropriate candidates who could omit surgery.

## Materials and methods

### Patients

This study assessed MRIs in a retrospective study population and applied the derived criteria in a prospective pilot study cohort. This study was approved by the institutional review board of National Cancer Center (NCC2017-0141, NCC2019-0276). For the retrospective analysis, the requirement for written informed consent was waived by the institutional review board of National Cancer Center. From November 2015 to March 2019, we identified 574 eligible patients who were diagnosed with stage II or III unilateral invasive breast cancer and underwent surgery after completion of NAC. Of these, 284 patients were excluded because of the following reasons: luminal subtype (n = 230, HR + HER2−), equivocal HER2 status (n = 19), lack of MRI after NAC completion (n = 25), and recurrent breast cancer (n = 10). Finally, 290 patients constituted the retrospective study population.

The prospective pilot study was designed to apply preliminary MRI diagnostic criteria and to identify any supplementary value of presurgical needle biopsies (needle biopsies after NAC and before surgery) for residual tumour detection. For this prospective part, written informed consent was obtained from all participants. From July 2017 to May 2018, 15 patients were enrolled in the prospective pilot study. Pre- and post-NAC MRI assessments were mandatory. The patients could choose whether to undergo a presurgical needle biopsy. The patients underwent an ultrasound-guided 14-gauge core needle biopsy at the tumour bed after completion of the NAC. Pre- and posttreatment MRI, ultrasound, and mammography data were comprehensively reviewed for the decision of the most appropriate biopsy site. One of two experienced breast radiologists obtained four to six tissue cores. The patients underwent surgery within 2 weeks of the date of the presurgical needle biopsy. The data of these patients were not included in the retrospective analysis.

### Clinical and histopathological data analysis

The medical records including patient age, pretreatment clinical TNM stage, NAC regimen, and operation record were reviewed. The initial diagnosis of breast cancer was based on an ultrasound-guided core needle biopsy. Routine histopathological reports included the histologic type and tumour grade. Pretreatment needle biopsy specimens were used for immunohistochemical assessment and subtype grouping. Negative oestrogen or progesterone receptor status was defined as fewer than 1% immunoreactive cells, respectively^24^. HER2 positivity was defined as membrane staining of 3+, whereas 1+ and 0 were defined as HER2-negative. Gene amplification using fluorescent in situ hybridization or dual-colour silver in situ hybridization was performed when the score was equivocal (2+). Tumours were classified into three subtypes as follows: HR-HER2- (TN), HR-HER2+, and HR+HER2+. Systemic treatment was administered at the discretion of the physician. Pathology after surgical excision was the reference standard for analysis. We defined breast pCR as the histopathologic absence of invasive tumour in removed breast specimens irrespective of remaining in situ lesions (ypT0/is)^25^.

### MRI acquisition and analysis

The following three MRI scanners were used with dedicated breast surface coils and the patient in a prone position: Signa HDxt 3.0 T (GE Healthcare, Milwaukee, WI), Achieva 3.0 T TX (Philips N.V., Eindhoven, The Netherlands), and Ingenia 3.0 T (Philips N.V.)^22^. MRI protocols consisted of the following sequences: an axial fat-suppressed T2-weighted sequence; a dynamic axial fat-suppressed T1-weighted sequence before and 90, 180, 270, and 360 s after an intravenous injection of gadoteric acid (0.2 mL/kg body weight, Dotarem; Guerbet, Aulnay-sous-Bois, France). The slice thickness of T1-weighted images was 2 mm. Subtraction and maximum intensity projection images were generated from the dynamic series. The median interval between post-NAC MRI and surgery was 4 days (range, 0 to 26 days).

All images were reviewed by a breast radiologist with 7 years of experience. The radiologist was blinded to the clinicopathological information except for the breast cancer diagnosis. When the post-NAC MRI was assessed, the pre-NAC MRI and other pre-NAC imaging examinations were reviewed for comparison. The BPE was classified as minimal, mild, moderate, and marked degree^26^. The lesion size and signal intensity (SI) were measured on early (first phase, 90 s after contrast agent injection) and late (last phase, 360 s after contrast agent injection) post-contrast T1-weighted images. The lesion size was defined as the maximal diameter of the enhancing lesion. The size was described as 0 cm if there was no contrast-enhanced lesion. The definition of the lesion-to-background parenchymal SER was a slight modification of the method used by Kim et al.^15^. The SER was defined as the SI of the lesion divided by the SI of the normal parenchyma of the contralateral breast. Two circular regions of interest with a median diameter of 2.2 mm (range, 1.8 to 3.1 mm) were manually drawn to include the highest enhancing portion of the tumour and the normal-appearing parenchyma. When an enhancing lesion disappeared after NAC, the SI of the lesion was measured at the site of the original tumour. The lesion size and SER on early- and late-phase images were defined as early size, late size, early SER, and late SER, respectively.

### Statistical analysis

Statistical analyses were performed using SAS version 9.4 (SAS Institute Inc., Cary, NC, USA) and R software, version 3.6.2 (R Project for Statistical Computing). The independent t-test, Wilcoxon rank-sum test, Fisher’s exact test, or Pearson’s chi-square test was used for comparisons between pCR and non-pCR patients, as appropriate. Receiver operating characteristic curves were built according to lesion size and SER in the early and late phases. Differences in AUCs were compared using the DeLong method. Multiple diagnostic performance parameters to identify a residual tumour including sensitivity, specificity, accuracy, positive predictive value (PPV), and NPV were calculated. The values were obtained in the overall population and in subgroups based on immunohistochemistry. A p-value < 0.05 was considered to be statistically significant.

Fifteen patients were eligible for the prospective assessment. As our main concern was to identify patients who can safely omit surgery, high sensitivity for detecting residual lesions and a high NPV (the probability that patients with rCR truly achieve pCR) were required. Therefore, we set the maximal MRI values as cut-off points, which shows over 90% sensitivity and NPV in this study.

### Ethical approval

All procedures performed in studies involving human participants were in accordance with the ethical standards of the institutional research committee of National Cancer Center (IRB No: NCC2017-0141, NCC2019-0276) and with the 1964 Helsinki Declaration and its later amendments or comparable ethical standards.

## Supplementary Information

Supplementary Information

## Data Availability

The datasets generated during and/or analysed during the current study are available from the corresponding author on reasonable request.
