# Supplementary material for: Criteria for identifying residual tumours after neoadjuvant chemotherapy of breast cancers: a magnetic resonance imaging study
Source: Sci Rep. 2021 Jan 12;11:634. doi: 10.1038/s41598-020-79743-8 (PMC7804856; doi:10.1038/s41598-020-79743-8)
Supplement: Supplementary file 1 — Supplementary Information [file 41598_2020_79743_MOESM1_ESM.pdf]

# Criteria for identifying residual tumours after neoadjuvant chemotherapy of breast cancers: a magnetic resonance imaging study

Author names

Yunju Kim, Sung Hoon Sim, Boram Park, In Hye Chae, Jai Hong Han, So-Youn Jung, Seeyoun Lee, Youngmi Kwon, In Hae Park, Kyounglan Ko, Chan Wha Lee, Keun Seok Lee, Han-Sung Kang, Eun Sook Lee

**Supplementary Figure.** The results of MRI assessments, presurgical needle biopsies, and final surgeries in HR-HER2+ or HR-HER- patients (n = 13). The HR+HER2+ patients (n = 2) were not included. *NAC* neoadjuvant chemotherapy, *MRI* magnetic resonance imaging, *HR* hormone receptor, *HER2* human epidermal growth factor receptor 2, *CNB* core needle biopsy, *SER* lesion-to-background parenchymal signal enhancement ratio, *rCR* radiological complete response, *pCR* pathological complete response, *ypT* post-NAC T stage

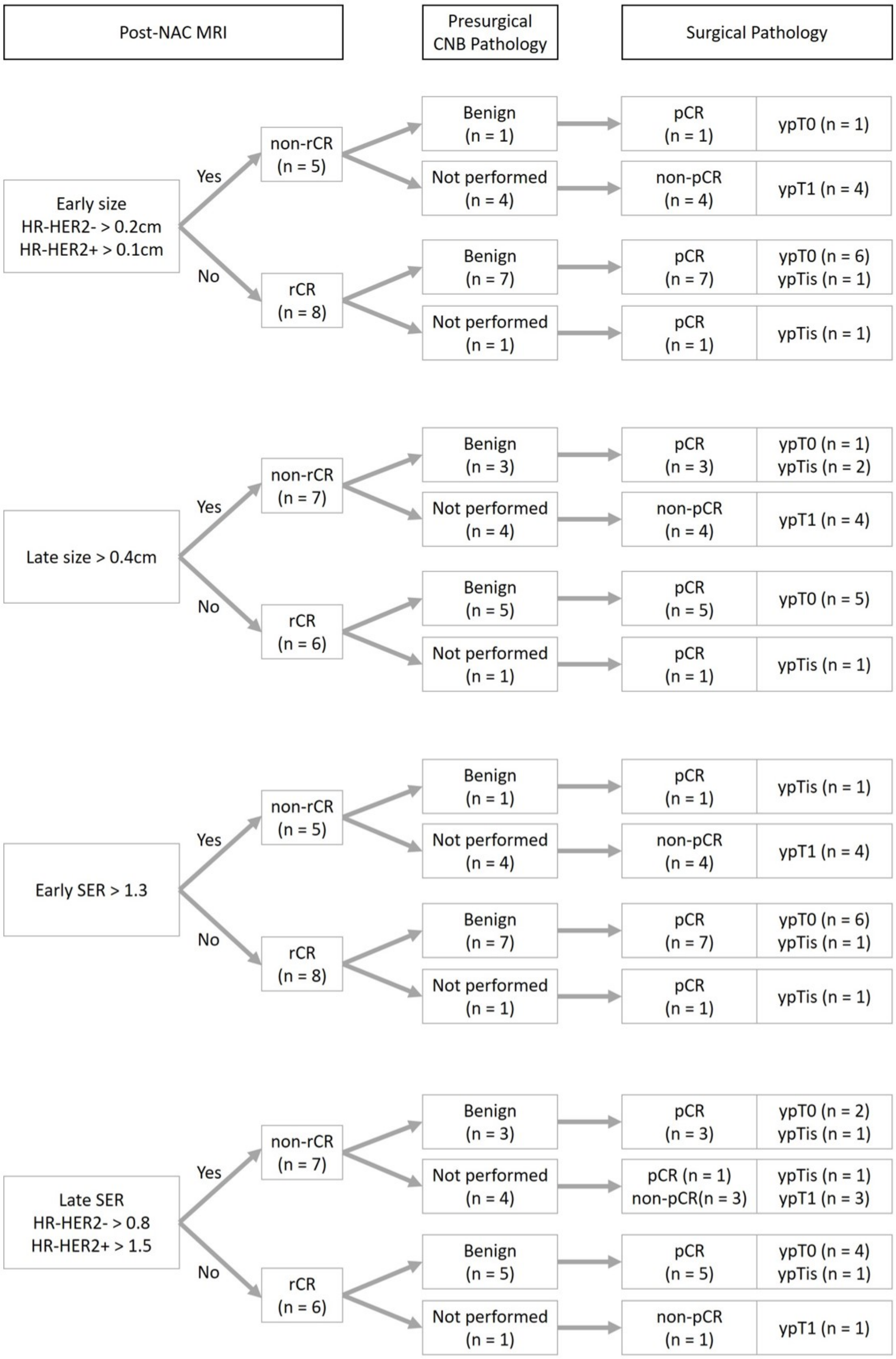

# Supplementary Table

**Contents**

**1. parameter definition**

**2. exploring cutoff values according to parameters in HR-HER2-, HR-HER2+ and HR+HER2+ subtype**

## 1. parameter definition

| | | |
|---|---|---|
| Correct | Event | TP (true positive) |
| Correct | Non-Envet | TN (true negative) |
| Incorrect | Event | FP (false positive) |
| Incorrect | Non-Envet | FN (false negative) |
| Percentages | Correct | (TP+TN)/total |
| Percentages | Sensitivity | TP/(TP+FN) |
| Percentages | Specificity | TN/(TN+FP) |
| Percentages | PPV | TP/(TP+FP) |
| Percentages | NPV | TN/(TN+FN) |

## 2. exploring cutoff values according to parameters in HR-HER2-, HR-HER2+ and HR+HER2+ subtype

### HR- HER2-

**MrE_Size: PostMR early phase (cm)**

| MrE_Size | Correct | | Incorrect | | Percentages | | | | |
|---|---|---|---|---|---|---|---|---|---|
| | Event | Non-Event | Event | Non-Event | Correct | Sensitivity | Specificity | PPV | NPV |
| 0 | 54 | 29 | 12 | 2 | 85.6% | 96.4% | 70.7% | 81.8% | 93.5% |
| 0.2 | 53 | 30 | 11 | 3 | 85.6% | 94.6% | 73.2% | 82.8% | 90.9% |
| 0.3 | 52 | 31 | 10 | 4 | 85.6% | 92.9% | 75.6% | 83.9% | 88.6% |
| 0.4 | 51 | 33 | 8 | 5 | 86.6% | 91.1% | 80.5% | 86.4% | 86.8% |
| 0.5 | 49 | 33 | 8 | 7 | 84.5% | 87.5% | 80.5% | 86.0% | 82.5% |
| 0.6 | 48 | 34 | 7 | 8 | 84.5% | 85.7% | 82.9% | 87.3% | 81.0% |
| 0.7 | 42 | 35 | 6 | 14 | 79.4% | 75.0% | 85.4% | 87.5% | 71.4% |
| 0.8 | 38 | 38 | 3 | 18 | 78.4% | 67.9% | 92.7% | 92.7% | 67.9% |
| 0.9 | 34 | 38 | 3 | 22 | 74.2% | 60.7% | 92.7% | 91.9% | 63.3% |
| 1 | 33 | 39 | 2 | 23 | 74.2% | 58.9% | 95.1% | 94.3% | 62.9% |
| 1.1 | 31 | 39 | 2 | 25 | 72.2% | 55.4% | 95.1% | 93.9% | 60.9% |
| 1.2 | 31 | 40 | 1 | 25 | 73.2% | 55.4% | 97.6% | 96.9% | 61.5% |
| 1.3 | 30 | 40 | 1 | 26 | 72.2% | 53.6% | 97.6% | 96.8% | 60.6% |
| 1.4 | 28 | 41 | 0 | 28 | 71.1% | 50.0% | 100.0% | 100.0% | 59.4% |
| 1.5 | 24 | 41 | 0 | 32 | 67.0% | 42.9% | 100.0% | 100.0% | 56.2% |
| 1.6 | 23 | 41 | 0 | 33 | 66.0% | 41.1% | 100.0% | 100.0% | 55.4% |
| 1.7 | 22 | 41 | 0 | 34 | 64.9% | 39.3% | 100.0% | 100.0% | 54.7% |
| 1.9 | 20 | 41 | 0 | 36 | 62.9% | 35.7% | 100.0% | 100.0% | 53.2% |
| 2 | 18 | 41 | 0 | 38 | 60.8% | 32.1% | 100.0% | 100.0% | 51.9% |
| 2.1 | 17 | 41 | 0 | 39 | 59.8% | 30.4% | 100.0% | 100.0% | 51.3% |
| 2.4 | 16 | 41 | 0 | 40 | 58.8% | 28.6% | 100.0% | 100.0% | 50.6% |
| 2.5 | 13 | 41 | 0 | 43 | 55.7% | 23.2% | 100.0% | 100.0% | 48.8% |
| 2.8 | 12 | 41 | 0 | 44 | 54.6% | 21.4% | 100.0% | 100.0% | 48.2% |
| 3 | 10 | 41 | 0 | 46 | 52.6% | 17.9% | 100.0% | 100.0% | 47.1% |
| 3.1 | 8 | 41 | 0 | 48 | 50.5% | 14.3% | 100.0% | 100.0% | 46.1% |
| 3.4 | 6 | 41 | 0 | 50 | 48.5% | 10.7% | 100.0% | 100.0% | 45.1% |
| 4 | 4 | 41 | 0 | 52 | 46.4% | 7.1% | 100.0% | 100.0% | 44.1% |
| 4.3 | 3 | 41 | 0 | 53 | 45.4% | 5.4% | 100.0% | 100.0% | 43.6% |
| 5 | 2 | 41 | 0 | 54 | 44.3% | 3.6% | 100.0% | 100.0% | 43.2% |
| 5.4 | 1 | 41 | 0 | 55 | 43.3% | 1.8% | 100.0% | 100.0% | 42.7% |
| 9.4 | 0 | 41 | 0 | 56 | 42.3% | 0.0% | 100.0% | NA | 42.3% |

**MrL_Size: PostMR late phase (cm)**

| MrL_Size | Correct | | Incorrect | | Percentages | | | | |
|---|---|---|---|---|---|---|---|---|---|
| | Event | Non-Event | Event | Non-Event | Correct | Sensitivity | Specificity | PPV | NPV |
| 0 | 56 | 26 | 15 | 0 | 84.5% | 100.0% | 63.4% | 78.9% | 100.0% |
| 0.2 | 56 | 27 | 14 | 0 | 85.6% | 100.0% | 65.9% | 80.0% | 100.0% |
| 0.4 | 54 | 30 | 11 | 2 | 86.6% | 96.4% | 73.2% | 83.1% | 93.8% |
| 0.5 | 52 | 30 | 11 | 4 | 84.5% | 92.9% | 73.2% | 82.5% | 88.2% |
| 0.6 | 52 | 32 | 9 | 4 | 86.6% | 92.9% | 78.0% | 85.2% | 88.9% |
| 0.7 | 48 | 32 | 9 | 8 | 82.5% | 85.7% | 78.0% | 84.2% | 80.0% |
| 0.8 | 42 | 34 | 7 | 14 | 78.4% | 75.0% | 82.9% | 85.7% | 70.8% |
| 0.9 | 39 | 34 | 7 | 17 | 75.3% | 69.6% | 82.9% | 84.8% | 66.7% |
| 1 | 37 | 36 | 5 | 19 | 75.3% | 66.1% | 87.8% | 88.1% | 65.5% |
| 1.1 | 34 | 37 | 4 | 22 | 73.2% | 60.7% | 90.2% | 89.5% | 62.7% |
| 1.2 | 33 | 38 | 3 | 23 | 73.2% | 58.9% | 92.7% | 91.7% | 62.3% |
| 1.3 | 32 | 39 | 2 | 24 | 73.2% | 57.1% | 95.1% | 94.1% | 61.9% |
| 1.4 | 31 | 40 | 1 | 25 | 73.2% | 55.4% | 97.6% | 96.9% | 61.5% |
| 1.5 | 26 | 41 | 0 | 30 | 69.1% | 46.4% | 100.0% | 100.0% | 57.7% |
| 1.6 | 24 | 41 | 0 | 32 | 67.0% | 42.9% | 100.0% | 100.0% | 56.2% |
| 1.7 | 23 | 41 | 0 | 33 | 66.0% | 41.1% | 100.0% | 100.0% | 55.4% |
| 1.9 | 21 | 41 | 0 | 35 | 63.9% | 37.5% | 100.0% | 100.0% | 53.9% |
| 2 | 19 | 41 | 0 | 37 | 61.9% | 33.9% | 100.0% | 100.0% | 52.6% |
| 2.1 | 18 | 41 | 0 | 38 | 60.8% | 32.1% | 100.0% | 100.0% | 51.9% |
| 2.2 | 17 | 41 | 0 | 39 | 59.8% | 30.4% | 100.0% | 100.0% | 51.3% |
| 2.4 | 16 | 41 | 0 | 40 | 58.8% | 28.6% | 100.0% | 100.0% | 50.6% |
| 2.5 | 13 | 41 | 0 | 43 | 55.7% | 23.2% | 100.0% | 100.0% | 48.8% |
| 2.8 | 12 | 41 | 0 | 44 | 54.6% | 21.4% | 100.0% | 100.0% | 48.2% |
| 3 | 10 | 41 | 0 | 46 | 52.6% | 17.9% | 100.0% | 100.0% | 47.1% |

| | | | | | | | | | |
|---|---|---|---|---|---|---|---|---|---|
| 3.1 | 8 | 41 | 0 | 48 | 50.5% | 14.3% | 100.0% | 100.0% | 46.1% |
| 3.4 | 6 | 41 | 0 | 50 | 48.5% | 10.7% | 100.0% | 100.0% | 45.1% |
| 4 | 4 | 41 | 0 | 52 | 46.4% | 7.1% | 100.0% | 100.0% | 44.1% |
| 4.3 | 3 | 41 | 0 | 53 | 45.4% | 5.4% | 100.0% | 100.0% | 43.6% |
| 5 | 2 | 41 | 0 | 54 | 44.3% | 3.6% | 100.0% | 100.0% | 43.2% |
| 5.4 | 1 | 41 | 0 | 55 | 43.3% | 1.8% | 100.0% | 100.0% | 42.7% |
| 9.4 | 0 | 41 | 0 | 56 | 42.3% | 0.0% | 100.0% | NA | 42.3% |

**MrSerE: Lesion-to-background SER (early)**

| MrSerE | Correct | | Incorrect | | Percentages | | | | |
|---|---|---|---|---|---|---|---|---|---|
| | Event | Non-Event | Event | Non-Event | Correct | Sensitivity | Specificity | PPV | NPV |
| 0.54347826 | 56 | 1 | 40 | 0 | 58.8% | 100.0% | 2.4% | 58.3% | 100.0% |
| 0.69809428 | 56 | 2 | 39 | 0 | 59.8% | 100.0% | 4.9% | 58.9% | 100.0% |
| 0.72930464 | 56 | 3 | 38 | 0 | 60.8% | 100.0% | 7.3% | 59.6% | 100.0% |
| 0.76327434 | 56 | 4 | 37 | 0 | 61.9% | 100.0% | 9.8% | 60.2% | 100.0% |
| 0.79584463 | 56 | 5 | 36 | 0 | 62.9% | 100.0% | 12.2% | 60.9% | 100.0% |
| 0.85960591 | 56 | 6 | 35 | 0 | 63.9% | 100.0% | 14.6% | 61.5% | 100.0% |
| 0.8875523 | 56 | 7 | 34 | 0 | 64.9% | 100.0% | 17.1% | 62.2% | 100.0% |
| 0.92962702 | 56 | 8 | 33 | 0 | 66.0% | 100.0% | 19.5% | 62.9% | 100.0% |
| 0.95883941 | 55 | 8 | 33 | 1 | 64.9% | 98.2% | 19.5% | 62.5% | 88.9% |
| 0.96180396 | 55 | 9 | 32 | 1 | 66.0% | 98.2% | 22.0% | 63.2% | 90.0% |
| 0.965 | 55 | 10 | 31 | 1 | 67.0% | 98.2% | 24.4% | 64.0% | 90.9% |
| 0.96806812 | 55 | 11 | 30 | 1 | 68.0% | 98.2% | 26.8% | 64.7% | 91.7% |
| 0.97256858 | 54 | 11 | 30 | 2 | 67.0% | 96.4% | 26.8% | 64.3% | 84.6% |
| 0.97966632 | 54 | 12 | 29 | 2 | 68.0% | 96.4% | 29.3% | 65.1% | 85.7% |
| 0.98854337 | 54 | 13 | 28 | 2 | 69.1% | 96.4% | 31.7% | 65.9% | 86.7% |
| 1.00252951 | 54 | 14 | 27 | 2 | 70.1% | 96.4% | 34.1% | 66.7% | 87.5% |
| 1.01390645 | 54 | 15 | 26 | 2 | 71.1% | 96.4% | 36.6% | 67.5% | 88.2% |
| 1.02138643 | 54 | 16 | 25 | 2 | 72.2% | 96.4% | 39.0% | 68.4% | 88.9% |
| 1.02740864 | 54 | 17 | 24 | 2 | 73.2% | 96.4% | 41.5% | 69.2% | 89.5% |
| 1.03007519 | 54 | 18 | 23 | 2 | 74.2% | 96.4% | 43.9% | 70.1% | 90.0% |
| 1.03414314 | 54 | 19 | 22 | 2 | 75.3% | 96.4% | 46.3% | 71.1% | 90.5% |
| 1.03637612 | 54 | 20 | 21 | 2 | 76.3% | 96.4% | 48.8% | 72.0% | 90.9% |
| 1.05256065 | 54 | 21 | 20 | 2 | 77.3% | 96.4% | 51.2% | 73.0% | 91.3% |
| 1.08962264 | 54 | 22 | 19 | 2 | 78.4% | 96.4% | 53.7% | 74.0% | 91.7% |
| 1.10700637 | 54 | 23 | 18 | 2 | 79.4% | 96.4% | 56.1% | 75.0% | 92.0% |
| 1.12696942 | 54 | 24 | 17 | 2 | 80.4% | 96.4% | 58.5% | 76.1% | 92.3% |
| 1.13831089 | 54 | 25 | 16 | 2 | 81.4% | 96.4% | 61.0% | 77.1% | 92.6% |
| 1.15317559 | 54 | 26 | 15 | 2 | 82.5% | 96.4% | 63.4% | 78.3% | 92.9% |
| 1.17159337 | 54 | 27 | 14 | 2 | 83.5% | 96.4% | 65.9% | 79.4% | 93.1% |
| 1.20485175 | 54 | 28 | 13 | 2 | 84.5% | 96.4% | 68.3% | 80.6% | 93.3% |
| 1.26836435 | 54 | 29 | 12 | 2 | 85.6% | 96.4% | 70.7% | 81.8% | 93.5% |
| 1.30039788 | 53 | 29 | 12 | 3 | 84.5% | 94.6% | 70.7% | 81.5% | 90.6% |
| 1.37086093 | 52 | 29 | 12 | 4 | 83.5% | 92.9% | 70.7% | 81.3% | 87.9% |
| 1.38021638 | 51 | 29 | 12 | 5 | 82.5% | 91.1% | 70.7% | 81.0% | 85.3% |
| 1.45962733 | 50 | 29 | 12 | 6 | 81.4% | 89.3% | 70.7% | 80.6% | 82.9% |
| 1.6175359 | 50 | 30 | 11 | 6 | 82.5% | 89.3% | 73.2% | 82.0% | 83.3% |
| 1.70023148 | 50 | 31 | 10 | 6 | 83.5% | 89.3% | 75.6% | 83.3% | 83.8% |
| 1.80104031 | 49 | 31 | 10 | 7 | 82.5% | 87.5% | 75.6% | 83.1% | 81.6% |
| 1.8250774 | 49 | 32 | 9 | 7 | 83.5% | 87.5% | 78.0% | 84.5% | 82.1% |
| 1.91492865 | 49 | 33 | 8 | 7 | 84.5% | 87.5% | 80.5% | 86.0% | 82.5% |
| 1.92822967 | 48 | 33 | 8 | 8 | 83.5% | 85.7% | 80.5% | 85.7% | 80.5% |
| 1.94577554 | 48 | 34 | 7 | 8 | 84.5% | 85.7% | 82.9% | 87.3% | 81.0% |
| 1.95972115 | 48 | 35 | 6 | 8 | 85.6% | 85.7% | 85.4% | 88.9% | 81.4% |
| 2.01458333 | 47 | 35 | 6 | 9 | 84.5% | 83.9% | 85.4% | 88.7% | 79.5% |
| 2.02776025 | 46 | 35 | 6 | 10 | 83.5% | 82.1% | 85.4% | 88.5% | 77.8% |
| 2.06777317 | 45 | 35 | 6 | 11 | 82.5% | 80.4% | 85.4% | 88.2% | 76.1% |
| 2.08779631 | 44 | 35 | 6 | 12 | 81.4% | 78.6% | 85.4% | 88.0% | 74.5% |
| 2.13172805 | 43 | 35 | 6 | 13 | 80.4% | 76.8% | 85.4% | 87.8% | 72.9% |
| 2.15911731 | 42 | 35 | 6 | 14 | 79.4% | 75.0% | 85.4% | 87.5% | 71.4% |
| 2.16375087 | 41 | 35 | 6 | 15 | 78.4% | 73.2% | 85.4% | 87.2% | 70.0% |
| 2.17069701 | 40 | 35 | 6 | 16 | 77.3% | 71.4% | 85.4% | 87.0% | 68.6% |
| 2.1788953 | 39 | 35 | 6 | 17 | 76.3% | 69.6% | 85.4% | 86.7% | 67.3% |
| 2.18123275 | 38 | 35 | 6 | 18 | 75.3% | 67.9% | 85.4% | 86.4% | 66.0% |
| 2.19175515 | 38 | 36 | 5 | 18 | 76.3% | 67.9% | 87.8% | 88.4% | 66.7% |
| 2.21072797 | 37 | 36 | 5 | 19 | 75.3% | 66.1% | 87.8% | 88.1% | 65.5% |
| 2.2107438 | 36 | 36 | 5 | 20 | 74.2% | 64.3% | 87.8% | 87.8% | 64.3% |
| 2.26721763 | 35 | 36 | 5 | 21 | 73.2% | 62.5% | 87.8% | 87.5% | 63.2% |
| 2.28350515 | 34 | 36 | 5 | 22 | 72.2% | 60.7% | 87.8% | 87.2% | 62.1% |
| 2.29531873 | 33 | 36 | 5 | 23 | 71.1% | 58.9% | 87.8% | 86.8% | 61.0% |
| 2.30360721 | 33 | 37 | 4 | 23 | 72.2% | 58.9% | 90.2% | 89.2% | 61.7% |
| 2.3194707 | 32 | 37 | 4 | 24 | 71.1% | 57.1% | 90.2% | 88.9% | 60.7% |
| 2.32530864 | 31 | 37 | 4 | 25 | 70.1% | 55.4% | 90.2% | 88.6% | 59.7% |
| 2.35752577 | 30 | 37 | 4 | 26 | 69.1% | 53.6% | 90.2% | 88.2% | 58.7% |
| 2.38945006 | 30 | 38 | 3 | 26 | 70.1% | 53.6% | 92.7% | 90.9% | 59.4% |
| 2.44245927 | 29 | 38 | 3 | 27 | 69.1% | 51.8% | 92.7% | 90.6% | 58.5% |
| 2.55051813 | 28 | 38 | 3 | 28 | 68.0% | 50.0% | 92.7% | 90.3% | 57.6% |
| 2.58493353 | 27 | 38 | 3 | 29 | 67.0% | 48.2% | 92.7% | 90.0% | 56.7% |
| 2.68029529 | 26 | 38 | 3 | 30 | 66.0% | 46.4% | 92.7% | 89.7% | 55.9% |
| 2.81205674 | 25 | 38 | 3 | 31 | 64.9% | 44.6% | 92.7% | 89.3% | 55.1% |
| 2.8548124 | 24 | 38 | 3 | 32 | 63.9% | 42.9% | 92.7% | 88.9% | 54.3% |
| 2.91312559 | 24 | 39 | 2 | 32 | 64.9% | 42.9% | 95.1% | 92.3% | 54.9% |
| 2.96940195 | 23 | 39 | 2 | 33 | 63.9% | 41.1% | 95.1% | 92.0% | 54.2% |
| 3.14722222 | 22 | 39 | 2 | 34 | 62.9% | 39.3% | 95.1% | 91.7% | 53.4% |
| 3.15320911 | 21 | 39 | 2 | 35 | 61.9% | 37.5% | 95.1% | 91.3% | 52.7% |
| 3.21814672 | 20 | 39 | 2 | 36 | 60.8% | 35.7% | 95.1% | 90.9% | 52.0% |
| 3.27943615 | 19 | 39 | 2 | 37 | 59.8% | 33.9% | 95.1% | 90.5% | 51.3% |
| 3.29361702 | 18 | 39 | 2 | 38 | 58.8% | 32.1% | 95.1% | 90.0% | 50.6% |
| 3.3477918 | 17 | 39 | 2 | 39 | 57.7% | 30.4% | 95.1% | 89.5% | 50.0% |
| 3.35467565 | 17 | 40 | 1 | 39 | 58.8% | 30.4% | 97.6% | 94.4% | 50.6% |
| 3.3799448 | 17 | 41 | 0 | 39 | 59.8% | 30.4% | 100.0% | 100.0% | 51.3% |
| 3.44490075 | 16 | 41 | 0 | 40 | 58.8% | 28.6% | 100.0% | 100.0% | 50.6% |
| 3.51712329 | 15 | 41 | 0 | 41 | 57.7% | 26.8% | 100.0% | 100.0% | 50.0% |
| 3.54462243 | 14 | 41 | 0 | 42 | 56.7% | 25.0% | 100.0% | 100.0% | 49.4% |
| 3.61805054 | 13 | 41 | 0 | 43 | 55.7% | 23.2% | 100.0% | 100.0% | 48.8% |
| 3.71040724 | 12 | 41 | 0 | 44 | 54.6% | 21.4% | 100.0% | 100.0% | 48.2% |

| | | | | | | | | | |
|---|---|---|---|---|---|---|---|---|---|
| 3.72154964 | 11 | 41 | 0 | 45 | 53.6% | 19.6% | 100.0% | 100.0% | 47.7% |
| 3.72386224 | 10 | 41 | 0 | 46 | 52.6% | 17.9% | 100.0% | 100.0% | 47.1% |
| 3.77081192 | 9 | 41 | 0 | 47 | 51.5% | 16.1% | 100.0% | 100.0% | 46.6% |
| 3.88263763 | 8 | 41 | 0 | 48 | 50.5% | 14.3% | 100.0% | 100.0% | 46.1% |
| 4.4291642 | 7 | 41 | 0 | 49 | 49.5% | 12.5% | 100.0% | 100.0% | 45.6% |
| 4.58542141 | 6 | 41 | 0 | 50 | 48.5% | 10.7% | 100.0% | 100.0% | 45.1% |
| 4.915 | 5 | 41 | 0 | 51 | 47.4% | 8.9% | 100.0% | 100.0% | 44.6% |
| 5.02927722 | 4 | 41 | 0 | 52 | 46.4% | 7.1% | 100.0% | 100.0% | 44.1% |
| 5.11569149 | 3 | 41 | 0 | 53 | 45.4% | 5.4% | 100.0% | 100.0% | 43.6% |
| 5.26149425 | 2 | 41 | 0 | 54 | 44.3% | 3.6% | 100.0% | 100.0% | 43.2% |
| 5.34678299 | 1 | 41 | 0 | 55 | 43.3% | 1.8% | 100.0% | 100.0% | 42.7% |
| 7.29642058 | 0 | 41 | 0 | 56 | 42.3% | 0.0% | 100.0% | NA | 42.3% |

**MrSerL: Lesion-to-background SER (late)**

| MrSerL | Correct | | Incorrect | | Percentages | | | | |
|---|---|---|---|---|---|---|---|---|---|
| | Event | Non-Event | Event | Non-Event | Correct | Sensitivity | Specificity | PPV | NPV |
| 0.65033784 | 56 | 1 | 40 | 0 | 58.8% | 100.0% | 2.4% | 58.3% | 100.0% |
| 0.71527778 | 56 | 2 | 39 | 0 | 59.8% | 100.0% | 4.9% | 58.9% | 100.0% |
| 0.72189349 | 56 | 3 | 38 | 0 | 60.8% | 100.0% | 7.3% | 59.6% | 100.0% |
| 0.88482075 | 56 | 4 | 37 | 0 | 61.9% | 100.0% | 9.8% | 60.2% | 100.0% |
| 0.88629738 | 56 | 5 | 36 | 0 | 62.9% | 100.0% | 12.2% | 60.9% | 100.0% |
| 0.89071038 | 55 | 5 | 36 | 1 | 61.9% | 98.2% | 12.2% | 60.4% | 83.3% |
| 0.96013168 | 55 | 6 | 35 | 1 | 62.9% | 98.2% | 14.6% | 61.1% | 85.7% |
| 0.968 | 55 | 7 | 34 | 1 | 63.9% | 98.2% | 17.1% | 61.8% | 87.5% |
| 0.96844521 | 55 | 8 | 33 | 1 | 64.9% | 98.2% | 19.5% | 62.5% | 88.9% |
| 0.97962382 | 55 | 9 | 32 | 1 | 66.0% | 98.2% | 22.0% | 63.2% | 90.0% |
| 0.97989276 | 54 | 9 | 32 | 2 | 64.9% | 96.4% | 22.0% | 62.8% | 81.8% |
| 0.98768473 | 54 | 10 | 31 | 2 | 66.0% | 96.4% | 24.4% | 63.5% | 83.3% |
| 0.99179537 | 54 | 11 | 30 | 2 | 67.0% | 96.4% | 26.8% | 64.3% | 84.6% |
| 0.99834071 | 54 | 12 | 29 | 2 | 68.0% | 96.4% | 29.3% | 65.1% | 85.7% |
| 0.99889868 | 54 | 13 | 28 | 2 | 69.1% | 96.4% | 31.7% | 65.9% | 86.7% |
| 1.02497809 | 54 | 14 | 27 | 2 | 70.1% | 96.4% | 34.1% | 66.7% | 87.5% |
| 1.03524673 | 54 | 15 | 26 | 2 | 71.1% | 96.4% | 36.6% | 67.5% | 88.2% |
| 1.0361991 | 54 | 16 | 25 | 2 | 72.2% | 96.4% | 39.0% | 68.4% | 88.9% |
| 1.05216693 | 54 | 17 | 24 | 2 | 73.2% | 96.4% | 41.5% | 69.2% | 89.5% |
| 1.05474665 | 54 | 18 | 23 | 2 | 74.2% | 96.4% | 43.9% | 70.1% | 90.0% |
| 1.07093822 | 53 | 18 | 23 | 3 | 73.2% | 94.6% | 43.9% | 69.7% | 85.7% |
| 1.08 | 53 | 19 | 22 | 3 | 74.2% | 94.6% | 46.3% | 70.7% | 86.4% |
| 1.10779596 | 53 | 20 | 21 | 3 | 75.3% | 94.6% | 48.8% | 71.6% | 87.0% |
| 1.1122291 | 53 | 21 | 20 | 3 | 76.3% | 94.6% | 51.2% | 72.6% | 87.5% |
| 1.13010753 | 53 | 22 | 19 | 3 | 77.3% | 94.6% | 53.7% | 73.6% | 88.0% |
| 1.14096186 | 53 | 23 | 18 | 3 | 78.4% | 94.6% | 56.1% | 74.6% | 88.5% |
| 1.1723356 | 53 | 24 | 17 | 3 | 79.4% | 94.6% | 58.5% | 75.7% | 88.9% |
| 1.17573964 | 53 | 25 | 16 | 3 | 80.4% | 94.6% | 61.0% | 76.8% | 89.3% |
| 1.20591233 | 53 | 26 | 15 | 3 | 81.4% | 94.6% | 63.4% | 77.9% | 89.7% |
| 1.2074928 | 52 | 26 | 15 | 4 | 80.4% | 92.9% | 63.4% | 77.6% | 86.7% |
| 1.23667712 | 52 | 27 | 14 | 4 | 81.4% | 92.9% | 65.9% | 78.8% | 87.1% |
| 1.28153565 | 51 | 27 | 14 | 5 | 80.4% | 91.1% | 65.9% | 78.5% | 84.4% |
| 1.31318681 | 51 | 28 | 13 | 5 | 81.4% | 91.1% | 68.3% | 79.7% | 84.8% |
| 1.46085011 | 50 | 28 | 13 | 6 | 80.4% | 89.3% | 68.3% | 79.4% | 82.4% |
| 1.53846154 | 50 | 29 | 12 | 6 | 81.4% | 89.3% | 70.7% | 80.6% | 82.9% |
| 1.60465116 | 49 | 29 | 12 | 7 | 80.4% | 87.5% | 70.7% | 80.3% | 80.6% |
| 1.62455304 | 48 | 29 | 12 | 8 | 79.4% | 85.7% | 70.7% | 80.0% | 78.4% |
| 1.64071637 | 48 | 30 | 11 | 8 | 80.4% | 85.7% | 73.2% | 81.4% | 78.9% |
| 1.64220183 | 47 | 30 | 11 | 9 | 79.4% | 83.9% | 73.2% | 81.0% | 76.9% |
| 1.66361974 | 46 | 30 | 11 | 10 | 78.4% | 82.1% | 73.2% | 80.7% | 75.0% |
| 1.67409949 | 46 | 31 | 10 | 10 | 79.4% | 82.1% | 75.6% | 82.1% | 75.6% |
| 1.67763158 | 45 | 31 | 10 | 11 | 78.4% | 80.4% | 75.6% | 81.8% | 73.8% |
| 1.68041237 | 44 | 31 | 10 | 12 | 77.3% | 78.6% | 75.6% | 81.5% | 72.1% |
| 1.68230818 | 44 | 32 | 9 | 12 | 78.4% | 78.6% | 78.0% | 83.0% | 72.7% |
| 1.68658699 | 43 | 32 | 9 | 13 | 77.3% | 76.8% | 78.0% | 82.7% | 71.1% |
| 1.71275168 | 42 | 32 | 9 | 14 | 76.3% | 75.0% | 78.0% | 82.4% | 69.6% |
| 1.77463863 | 41 | 32 | 9 | 15 | 75.3% | 73.2% | 78.0% | 82.0% | 68.1% |
| 1.79509632 | 40 | 32 | 9 | 16 | 74.2% | 71.4% | 78.0% | 81.6% | 66.7% |
| 1.83136531 | 39 | 32 | 9 | 17 | 73.2% | 69.6% | 78.0% | 81.3% | 65.3% |
| 1.83659109 | 38 | 32 | 9 | 18 | 72.2% | 67.9% | 78.0% | 80.9% | 64.0% |
| 1.88789683 | 37 | 32 | 9 | 19 | 71.1% | 66.1% | 78.0% | 80.4% | 62.7% |
| 1.89448819 | 37 | 33 | 8 | 19 | 72.2% | 66.1% | 80.5% | 82.2% | 63.5% |
| 1.89970501 | 37 | 34 | 7 | 19 | 73.2% | 66.1% | 82.9% | 84.1% | 64.2% |
| 1.91875 | 36 | 34 | 7 | 20 | 72.2% | 64.3% | 82.9% | 83.7% | 63.0% |
| 1.92947368 | 35 | 34 | 7 | 21 | 71.1% | 62.5% | 82.9% | 83.3% | 61.8% |
| 1.93510929 | 34 | 34 | 7 | 22 | 70.1% | 60.7% | 82.9% | 82.9% | 60.7% |
| 1.94023904 | 34 | 35 | 6 | 22 | 71.1% | 60.7% | 85.4% | 85.0% | 61.4% |
| 1.95302013 | 33 | 35 | 6 | 23 | 70.1% | 58.9% | 85.4% | 84.6% | 60.3% |
| 2.00977199 | 32 | 35 | 6 | 24 | 69.1% | 57.1% | 85.4% | 84.2% | 59.3% |
| 2.02723083 | 31 | 35 | 6 | 25 | 68.0% | 55.4% | 85.4% | 83.8% | 58.3% |
| 2.08108108 | 30 | 35 | 6 | 26 | 67.0% | 53.6% | 85.4% | 83.3% | 57.4% |
| 2.08336689 | 29 | 35 | 6 | 27 | 66.0% | 51.8% | 85.4% | 82.9% | 56.5% |
| 2.09867173 | 28 | 35 | 6 | 28 | 64.9% | 50.0% | 85.4% | 82.4% | 55.6% |
| 2.12016386 | 28 | 36 | 5 | 28 | 66.0% | 50.0% | 87.8% | 84.8% | 56.3% |
| 2.17551463 | 27 | 36 | 5 | 29 | 64.9% | 48.2% | 87.8% | 84.4% | 55.4% |
| 2.20685901 | 26 | 36 | 5 | 30 | 63.9% | 46.4% | 87.8% | 83.9% | 54.5% |
| 2.21086262 | 25 | 36 | 5 | 31 | 62.9% | 44.6% | 87.8% | 83.3% | 53.7% |
| 2.2140056 | 24 | 36 | 5 | 32 | 61.9% | 42.9% | 87.8% | 82.8% | 52.9% |
| 2.21947054 | 23 | 36 | 5 | 33 | 60.8% | 41.1% | 87.8% | 82.1% | 52.2% |
| 2.22570726 | 23 | 37 | 4 | 33 | 61.9% | 41.1% | 90.2% | 85.2% | 52.9% |
| 2.28246319 | 22 | 37 | 4 | 34 | 60.8% | 39.3% | 90.2% | 84.6% | 52.1% |
| 2.31334459 | 21 | 37 | 4 | 35 | 59.8% | 37.5% | 90.2% | 84.0% | 51.4% |
| 2.32785661 | 21 | 38 | 3 | 35 | 60.8% | 37.5% | 92.7% | 87.5% | 52.1% |
| 2.33542977 | 20 | 38 | 3 | 36 | 59.8% | 35.7% | 92.7% | 87.0% | 51.4% |
| 2.35182534 | 19 | 38 | 3 | 37 | 58.8% | 33.9% | 92.7% | 86.4% | 50.7% |
| 2.42398649 | 18 | 38 | 3 | 38 | 57.7% | 32.1% | 92.7% | 85.7% | 50.0% |
| 2.42978723 | 17 | 38 | 3 | 39 | 56.7% | 30.4% | 92.7% | 85.0% | 49.4% |
| 2.46713852 | 16 | 38 | 3 | 40 | 55.7% | 28.6% | 92.7% | 84.2% | 48.7% |
| 2.46794572 | 15 | 38 | 3 | 41 | 54.6% | 26.8% | 92.7% | 83.3% | 48.1% |
| 2.50455927 | 14 | 38 | 3 | 42 | 53.6% | 25.0% | 92.7% | 82.4% | 47.5% |
| 2.54775059 | 14 | 39 | 2 | 42 | 54.6% | 25.0% | 95.1% | 87.5% | 48.1% |
| 2.58223938 | 13 | 39 | 2 | 43 | 53.6% | 23.2% | 95.1% | 86.7% | 47.6% |
| 2.59341951 | 12 | 39 | 2 | 44 | 52.6% | 21.4% | 95.1% | 85.7% | 47.0% |

| | | | | | | | | | |
|---|---|---|---|---|---|---|---|---|---|
| 2.6519411 | 11 | 39 | 2 | 45 | 51.5% | 19.6% | 95.1% | 84.6% | 46.4% |
| 2.65326021 | 10 | 39 | 2 | 46 | 50.5% | 17.9% | 95.1% | 83.3% | 45.9% |
| 2.81408898 | 9 | 39 | 2 | 47 | 49.5% | 16.1% | 95.1% | 81.8% | 45.3% |
| 2.8484589 | 9 | 40 | 1 | 47 | 50.5% | 16.1% | 97.6% | 90.0% | 46.0% |
| 2.86087866 | 8 | 40 | 1 | 48 | 49.5% | 14.3% | 97.6% | 88.9% | 45.5% |
| 2.93694196 | 7 | 40 | 1 | 49 | 48.5% | 12.5% | 97.6% | 87.5% | 44.9% |
| 2.98645216 | 6 | 40 | 1 | 50 | 47.4% | 10.7% | 97.6% | 85.7% | 44.4% |
| 3.13879599 | 5 | 40 | 1 | 51 | 46.4% | 8.9% | 97.6% | 83.3% | 44.0% |
| 3.22093023 | 4 | 40 | 1 | 52 | 45.4% | 7.1% | 97.6% | 80.0% | 43.5% |
| 3.23469388 | 3 | 40 | 1 | 53 | 44.3% | 5.4% | 97.6% | 75.0% | 43.0% |
| 3.28313671 | 2 | 40 | 1 | 54 | 43.3% | 3.6% | 97.6% | 66.7% | 42.6% |
| 3.57936508 | 1 | 40 | 1 | 55 | 42.3% | 1.8% | 97.6% | 50.0% | 42.1% |
| 3.71049596 | 0 | 40 | 1 | 56 | 41.2% | 0.0% | 97.6% | 0.0% | 41.7% |
| 4.13732098 | 0 | 41 | 0 | 56 | 42.3% | 0.0% | 100.0% | NA | 42.3% |

# HR- HER2+

**MrE_Size: PostMR early phase (cm)**

| MrE_Size | Correct | | Incorrect | | Percentages | | | | |
|---|---|---|---|---|---|---|---|---|---|
| | Event | Non-Event | Event | Non-Event | Correct | Sensitivity | Specificity | PPV | NPV |
| 0.1 | 26 | 34 | 28 | 2 | 66.7% | 92.9% | 54.8% | 48.1% | 94.4% |
| 0.2 | 25 | 35 | 27 | 3 | 66.7% | 89.3% | 56.5% | 48.1% | 92.1% |
| 0.3 | 25 | 40 | 22 | 3 | 72.2% | 89.3% | 64.5% | 53.2% | 93.0% |
| 0.4 | 24 | 44 | 18 | 4 | 75.6% | 85.7% | 71.0% | 57.1% | 91.7% |
| 0.5 | 23 | 47 | 15 | 5 | 77.8% | 82.1% | 75.8% | 60.5% | 90.4% |
| 0.6 | 19 | 48 | 14 | 9 | 74.4% | 67.9% | 77.4% | 57.6% | 84.2% |
| 0.8 | 18 | 49 | 13 | 10 | 74.4% | 64.3% | 79.0% | 58.1% | 83.1% |
| 0.9 | 17 | 50 | 12 | 11 | 74.4% | 60.7% | 80.6% | 58.6% | 82.0% |
| 1 | 17 | 51 | 11 | 11 | 75.6% | 60.7% | 82.3% | 60.7% | 82.3% |
| 1.1 | 16 | 52 | 10 | 12 | 75.6% | 57.1% | 83.9% | 61.5% | 81.3% |
| 1.2 | 14 | 55 | 7 | 14 | 76.7% | 50.0% | 88.7% | 66.7% | 79.7% |
| 1.4 | 13 | 56 | 6 | 15 | 76.7% | 46.4% | 90.3% | 68.4% | 78.9% |
| 1.5 | 9 | 57 | 5 | 19 | 73.3% | 32.1% | 91.9% | 64.3% | 75.0% |
| 1.7 | 8 | 57 | 5 | 20 | 72.2% | 28.6% | 91.9% | 61.5% | 74.0% |
| 1.8 | 8 | 58 | 4 | 20 | 73.3% | 28.6% | 93.5% | 66.7% | 74.4% |
| 1.9 | 7 | 59 | 3 | 21 | 73.3% | 25.0% | 95.2% | 70.0% | 73.8% |
| 2 | 6 | 61 | 1 | 22 | 74.4% | 21.4% | 98.4% | 85.7% | 73.5% |
| 2.7 | 5 | 61 | 1 | 23 | 73.3% | 17.9% | 98.4% | 83.3% | 72.6% |
| 2.9 | 4 | 61 | 1 | 24 | 72.2% | 14.3% | 98.4% | 80.0% | 71.8% |
| 3.5 | 4 | 62 | 0 | 24 | 73.3% | 14.3% | 100.0% | 100.0% | 72.1% |
| 3.9 | 3 | 62 | 0 | 25 | 72.2% | 10.7% | 100.0% | 100.0% | 71.3% |
| 4.7 | 2 | 62 | 0 | 26 | 71.1% | 7.1% | 100.0% | 100.0% | 70.5% |
| 4.9 | 1 | 62 | 0 | 27 | 70.0% | 3.6% | 100.0% | 100.0% | 69.7% |
| 5 | 0 | 62 | 0 | 28 | 68.9% | 0.0% | 100.0% | NA | 68.9% |

**MrL_Size: PostMR late phase (cm)**

| MrL_Size | Correct | | Incorrect | | Percentages | | | | |
|---|---|---|---|---|---|---|---|---|---|
| | Event | Non-Event | Event | Non-Event | Correct | Sensitivity | Specificity | PPV | NPV |
| 0 | 27 | 29 | 33 | 1 | 62.2% | 96.4% | 46.8% | 45.0% | 96.7% |
| 0.3 | 26 | 31 | 31 | 2 | 63.3% | 92.9% | 50.0% | 45.6% | 93.9% |
| 0.4 | 26 | 38 | 24 | 2 | 71.1% | 92.9% | 61.3% | 52.0% | 95.0% |
| 0.5 | 25 | 40 | 22 | 3 | 72.2% | 89.3% | 64.5% | 53.2% | 93.0% |
| 0.6 | 24 | 40 | 22 | 4 | 71.1% | 85.7% | 64.5% | 52.2% | 90.9% |
| 0.7 | 22 | 42 | 20 | 6 | 71.1% | 78.6% | 67.7% | 52.4% | 87.5% |
| 0.8 | 21 | 42 | 20 | 7 | 70.0% | 75.0% | 67.7% | 51.2% | 85.7% |
| 0.9 | 20 | 43 | 19 | 8 | 70.0% | 71.4% | 69.4% | 51.3% | 84.3% |
| 1 | 18 | 44 | 18 | 10 | 68.9% | 64.3% | 71.0% | 50.0% | 81.5% |
| 1.2 | 16 | 47 | 15 | 12 | 70.0% | 57.1% | 75.8% | 51.6% | 79.7% |
| 1.3 | 16 | 49 | 13 | 12 | 72.2% | 57.1% | 79.0% | 55.2% | 80.3% |
| 1.4 | 14 | 51 | 11 | 14 | 72.2% | 50.0% | 82.3% | 56.0% | 78.5% |
| 1.5 | 9 | 52 | 10 | 19 | 67.8% | 32.1% | 83.9% | 47.4% | 73.2% |
| 1.7 | 8 | 52 | 10 | 20 | 66.7% | 28.6% | 83.9% | 44.4% | 72.2% |
| 1.9 | 7 | 53 | 9 | 21 | 66.7% | 25.0% | 85.5% | 43.8% | 71.6% |
| 2 | 6 | 57 | 5 | 22 | 70.0% | 21.4% | 91.9% | 54.5% | 72.2% |
| 2.1 | 6 | 58 | 4 | 22 | 71.1% | 21.4% | 93.5% | 60.0% | 72.5% |
| 2.5 | 6 | 60 | 2 | 22 | 73.3% | 21.4% | 96.8% | 75.0% | 73.2% |
| 2.7 | 5 | 60 | 2 | 23 | 72.2% | 17.9% | 96.8% | 71.4% | 72.3% |
| 2.8 | 5 | 61 | 1 | 23 | 73.3% | 17.9% | 98.4% | 83.3% | 72.6% |
| 2.9 | 4 | 61 | 1 | 24 | 72.2% | 14.3% | 98.4% | 80.0% | 71.8% |
| 3.5 | 4 | 62 | 0 | 24 | 73.3% | 14.3% | 100.0% | 100.0% | 72.1% |
| 4 | 3 | 62 | 0 | 25 | 72.2% | 10.7% | 100.0% | 100.0% | 71.3% |
| 4.7 | 2 | 62 | 0 | 26 | 71.1% | 7.1% | 100.0% | 100.0% | 70.5% |
| 4.9 | 1 | 62 | 0 | 27 | 70.0% | 3.6% | 100.0% | 100.0% | 69.7% |
| 5 | 0 | 62 | 0 | 28 | 68.9% | 0.0% | 100.0% | NA | 68.9% |

**MrSerE: Lesion-to-background SER (early)**

| MrSerE | Correct | | Incorrect | | Percentages | | | | |
|---|---|---|---|---|---|---|---|---|---|
| | Event | Non-Event | Event | Non-Event | Correct | Sensitivity | Specificity | PPV | NPV |
| 0.73229572 | 28 | 1 | 61 | 0 | 32.2% | 100.0% | 1.6% | 31.5% | 100.0% |
| 0.74043261 | 28 | 2 | 60 | 0 | 33.3% | 100.0% | 3.2% | 31.8% | 100.0% |
| 0.80313837 | 28 | 3 | 59 | 0 | 34.4% | 100.0% | 4.8% | 32.2% | 100.0% |

| | | | | | | | | | |
|---|---|---|---|---|---|---|---|---|---|
| 0.85235921 | 28 | 4 | 58 | 0 | 35.6% | 100.0% | 6.5% | 32.6% | 100.0% |
| 0.86666667 | 28 | 5 | 57 | 0 | 36.7% | 100.0% | 8.1% | 32.9% | 100.0% |
| 0.88995215 | 28 | 6 | 56 | 0 | 37.8% | 100.0% | 9.7% | 33.3% | 100.0% |
| 0.90132036 | 28 | 7 | 55 | 0 | 38.9% | 100.0% | 11.3% | 33.7% | 100.0% |
| 0.90861423 | 28 | 8 | 54 | 0 | 40.0% | 100.0% | 12.9% | 34.1% | 100.0% |
| 0.91207258 | 28 | 9 | 53 | 0 | 41.1% | 100.0% | 14.5% | 34.6% | 100.0% |
| 0.93038822 | 28 | 10 | 52 | 0 | 42.2% | 100.0% | 16.1% | 35.0% | 100.0% |
| 0.94070081 | 28 | 11 | 51 | 0 | 43.3% | 100.0% | 17.7% | 35.4% | 100.0% |
| 0.94097222 | 28 | 12 | 50 | 0 | 44.4% | 100.0% | 19.4% | 35.9% | 100.0% |
| 0.96033403 | 28 | 13 | 49 | 0 | 45.6% | 100.0% | 21.0% | 36.4% | 100.0% |
| 0.96722505 | 28 | 14 | 48 | 0 | 46.7% | 100.0% | 22.6% | 36.8% | 100.0% |
| 0.97254151 | 28 | 15 | 47 | 0 | 47.8% | 100.0% | 24.2% | 37.3% | 100.0% |
| 0.98539435 | 27 | 15 | 47 | 1 | 46.7% | 96.4% | 24.2% | 36.5% | 93.8% |
| 0.99953596 | 27 | 16 | 46 | 1 | 47.8% | 96.4% | 25.8% | 37.0% | 94.1% |
| 1.00679852 | 27 | 17 | 45 | 1 | 48.9% | 96.4% | 27.4% | 37.5% | 94.4% |
| 1.00788644 | 27 | 18 | 44 | 1 | 50.0% | 96.4% | 29.0% | 38.0% | 94.7% |
| 1.01697217 | 27 | 19 | 43 | 1 | 51.1% | 96.4% | 30.6% | 38.6% | 95.0% |
| 1.02249806 | 27 | 20 | 42 | 1 | 52.2% | 96.4% | 32.3% | 39.1% | 95.2% |
| 1.04861111 | 27 | 21 | 41 | 1 | 53.3% | 96.4% | 33.9% | 39.7% | 95.5% |
| 1.05626327 | 27 | 22 | 40 | 1 | 54.4% | 96.4% | 35.5% | 40.3% | 95.7% |
| 1.06462585 | 27 | 23 | 39 | 1 | 55.6% | 96.4% | 37.1% | 40.9% | 95.8% |
| 1.08531469 | 27 | 24 | 38 | 1 | 56.7% | 96.4% | 38.7% | 41.5% | 96.0% |
| 1.089838 | 27 | 25 | 37 | 1 | 57.8% | 96.4% | 40.3% | 42.2% | 96.2% |
| 1.0912 | 27 | 26 | 36 | 1 | 58.9% | 96.4% | 41.9% | 42.9% | 96.3% |
| 1.0976801 | 27 | 27 | 35 | 1 | 60.0% | 96.4% | 43.5% | 43.5% | 96.4% |
| 1.12529928 | 27 | 28 | 34 | 1 | 61.1% | 96.4% | 45.2% | 44.3% | 96.6% |
| 1.13662457 | 27 | 29 | 33 | 1 | 62.2% | 96.4% | 46.8% | 45.0% | 96.7% |
| 1.20144628 | 27 | 30 | 32 | 1 | 63.3% | 96.4% | 48.4% | 45.8% | 96.8% |
| 1.22770398 | 27 | 31 | 31 | 1 | 64.4% | 96.4% | 50.0% | 46.6% | 96.9% |
| 1.24012006 | 26 | 31 | 31 | 2 | 63.3% | 92.9% | 50.0% | 45.6% | 93.9% |
| 1.25413534 | 26 | 32 | 30 | 2 | 64.4% | 92.9% | 51.6% | 46.4% | 94.1% |
| 1.34912187 | 26 | 33 | 29 | 2 | 65.6% | 92.9% | 53.2% | 47.3% | 94.3% |
| 1.35621521 | 25 | 33 | 29 | 3 | 64.4% | 89.3% | 53.2% | 46.3% | 91.7% |
| 1.3718048 | 25 | 34 | 28 | 3 | 65.6% | 89.3% | 54.8% | 47.2% | 91.9% |
| 1.39849624 | 25 | 35 | 27 | 3 | 66.7% | 89.3% | 56.5% | 48.1% | 92.1% |
| 1.41333333 | 25 | 36 | 26 | 3 | 67.8% | 89.3% | 58.1% | 49.0% | 92.3% |
| 1.45472062 | 24 | 36 | 26 | 4 | 66.7% | 85.7% | 58.1% | 48.0% | 90.0% |
| 1.4640411 | 24 | 37 | 25 | 4 | 67.8% | 85.7% | 59.7% | 49.0% | 90.2% |
| 1.48255814 | 24 | 38 | 24 | 4 | 68.9% | 85.7% | 61.3% | 50.0% | 90.5% |
| 1.5 | 24 | 39 | 23 | 4 | 70.0% | 85.7% | 62.9% | 51.1% | 90.7% |
| 1.71376281 | 24 | 40 | 22 | 4 | 71.1% | 85.7% | 64.5% | 52.2% | 90.9% |
| 1.7185261 | 24 | 41 | 21 | 4 | 72.2% | 85.7% | 66.1% | 53.3% | 91.1% |
| 1.72035139 | 23 | 41 | 21 | 5 | 71.1% | 82.1% | 66.1% | 52.3% | 89.1% |
| 1.72812846 | 23 | 42 | 20 | 5 | 72.2% | 82.1% | 67.7% | 53.5% | 89.4% |
| 1.73112209 | 23 | 43 | 19 | 5 | 73.3% | 82.1% | 69.4% | 54.8% | 89.6% |
| 1.74562584 | 23 | 44 | 18 | 5 | 74.4% | 82.1% | 71.0% | 56.1% | 89.8% |
| 1.77871148 | 22 | 44 | 18 | 6 | 73.3% | 78.6% | 71.0% | 55.0% | 88.0% |
| 1.78716645 | 22 | 45 | 17 | 6 | 74.4% | 78.6% | 72.6% | 56.4% | 88.2% |
| 1.79499011 | 22 | 46 | 16 | 6 | 75.6% | 78.6% | 74.2% | 57.9% | 88.5% |
| 1.81463112 | 21 | 46 | 16 | 7 | 74.4% | 75.0% | 74.2% | 56.8% | 86.8% |
| 1.83275261 | 20 | 46 | 16 | 8 | 73.3% | 71.4% | 74.2% | 55.6% | 85.2% |
| 1.84267913 | 20 | 47 | 15 | 8 | 74.4% | 71.4% | 75.8% | 57.1% | 85.5% |
| 1.90349819 | 20 | 48 | 14 | 8 | 75.6% | 71.4% | 77.4% | 58.8% | 85.7% |
| 1.9040404 | 20 | 49 | 13 | 8 | 76.7% | 71.4% | 79.0% | 60.6% | 86.0% |
| 1.94574096 | 20 | 50 | 12 | 8 | 77.8% | 71.4% | 80.6% | 62.5% | 86.2% |
| 2.09027778 | 20 | 51 | 11 | 8 | 78.9% | 71.4% | 82.3% | 64.5% | 86.4% |
| 2.10784939 | 20 | 52 | 10 | 8 | 80.0% | 71.4% | 83.9% | 66.7% | 86.7% |
| 2.11422414 | 19 | 52 | 10 | 9 | 78.9% | 67.9% | 83.9% | 65.5% | 85.2% |
| 2.21338506 | 19 | 53 | 9 | 9 | 80.0% | 67.9% | 85.5% | 67.9% | 85.5% |
| 2.23391304 | 18 | 53 | 9 | 10 | 78.9% | 64.3% | 85.5% | 66.7% | 84.1% |
| 2.28843474 | 17 | 53 | 9 | 11 | 77.8% | 60.7% | 85.5% | 65.4% | 82.8% |
| 2.37558442 | 17 | 54 | 8 | 11 | 78.9% | 60.7% | 87.1% | 68.0% | 83.1% |
| 2.38088012 | 17 | 55 | 7 | 11 | 80.0% | 60.7% | 88.7% | 70.8% | 83.3% |
| 2.42731887 | 16 | 55 | 7 | 12 | 78.9% | 57.1% | 88.7% | 69.6% | 82.1% |
| 2.48684211 | 15 | 55 | 7 | 13 | 77.8% | 53.6% | 88.7% | 68.2% | 80.9% |
| 2.50484653 | 14 | 55 | 7 | 14 | 76.7% | 50.0% | 88.7% | 66.7% | 79.7% |
| 2.52373887 | 14 | 56 | 6 | 14 | 77.8% | 50.0% | 90.3% | 70.0% | 80.0% |
| 2.64129032 | 13 | 56 | 6 | 15 | 76.7% | 46.4% | 90.3% | 68.4% | 78.9% |
| 2.81043745 | 12 | 56 | 6 | 16 | 75.6% | 42.9% | 90.3% | 66.7% | 77.8% |
| 2.84210526 | 11 | 56 | 6 | 17 | 74.4% | 39.3% | 90.3% | 64.7% | 76.7% |
| 2.94699647 | 11 | 57 | 5 | 17 | 75.6% | 39.3% | 91.9% | 68.8% | 77.0% |
| 3.09090909 | 10 | 57 | 5 | 18 | 74.4% | 35.7% | 91.9% | 66.7% | 76.0% |
| 3.09097222 | 9 | 57 | 5 | 19 | 73.3% | 32.1% | 91.9% | 64.3% | 75.0% |
| 3.10046368 | 8 | 57 | 5 | 20 | 72.2% | 28.6% | 91.9% | 61.5% | 74.0% |
| 3.24074074 | 8 | 58 | 4 | 20 | 73.3% | 28.6% | 93.5% | 66.7% | 74.4% |
| 3.24349442 | 7 | 58 | 4 | 21 | 72.2% | 25.0% | 93.5% | 63.6% | 73.4% |
| 3.26987952 | 6 | 58 | 4 | 22 | 71.1% | 21.4% | 93.5% | 60.0% | 72.5% |
| 3.28061224 | 6 | 59 | 3 | 22 | 72.2% | 21.4% | 95.2% | 66.7% | 72.8% |
| 3.29191797 | 6 | 60 | 2 | 22 | 73.3% | 21.4% | 96.8% | 75.0% | 73.2% |
| 3.30882353 | 6 | 61 | 1 | 22 | 74.4% | 21.4% | 98.4% | 85.7% | 73.5% |
| 3.36605317 | 5 | 61 | 1 | 23 | 73.3% | 17.9% | 98.4% | 83.3% | 72.6% |
| 3.53604436 | 4 | 61 | 1 | 24 | 72.2% | 14.3% | 98.4% | 80.0% | 71.8% |
| 3.58419753 | 3 | 61 | 1 | 25 | 71.1% | 10.7% | 98.4% | 75.0% | 70.9% |
| 3.92924126 | 2 | 61 | 1 | 26 | 70.0% | 7.1% | 98.4% | 66.7% | 70.1% |
| 3.94477086 | 1 | 61 | 1 | 27 | 68.9% | 3.6% | 98.4% | 50.0% | 69.3% |
| 4.5746321 | 0 | 61 | 1 | 28 | 67.8% | 0.0% | 98.4% | 0.0% | 68.5% |
| 5.63157895 | 0 | 62 | 0 | 28 | 68.9% | 0.0% | 100.0% | NA | 68.9% |

**MrSerL: Lesion-to-background SER (late)**

| MrSerL | Correct | | Incorrect | | Percentages | | | | |
|---|---|---|---|---|---|---|---|---|---|
| | Event | Non-Event | Event | Non-Event | Correct | Sensitivity | Specificity | PPV | NPV |
| 0.63573668 | 28 | 1 | 61 | 0 | 32.2% | 100.0% | 1.6% | 31.5% | 100.0% |
| 0.72011057 | 28 | 2 | 60 | 0 | 33.3% | 100.0% | 3.2% | 31.8% | 100.0% |
| 0.79032258 | 28 | 3 | 59 | 0 | 34.4% | 100.0% | 4.8% | 32.2% | 100.0% |
| 0.79306072 | 28 | 4 | 58 | 0 | 35.6% | 100.0% | 6.5% | 32.6% | 100.0% |
| 0.86521483 | 28 | 5 | 57 | 0 | 36.7% | 100.0% | 8.1% | 32.9% | 100.0% |
| 0.88482633 | 28 | 6 | 56 | 0 | 37.8% | 100.0% | 9.7% | 33.3% | 100.0% |

| | | | | | | | | | |
|---|---|---|---|---|---|---|---|---|---|
| 0.90266729 | 28 | 7 | 55 | 0 | 38.9% | 100.0% | 11.3% | 33.7% | 100.0% |
| 0.90509554 | 28 | 8 | 54 | 0 | 40.0% | 100.0% | 12.9% | 34.1% | 100.0% |
| 0.91651432 | 28 | 9 | 53 | 0 | 41.1% | 100.0% | 14.5% | 34.6% | 100.0% |
| 0.93935719 | 28 | 10 | 52 | 0 | 42.2% | 100.0% | 16.1% | 35.0% | 100.0% |
| 0.95783133 | 27 | 10 | 52 | 1 | 41.1% | 96.4% | 16.1% | 34.2% | 90.9% |
| 0.98103976 | 27 | 11 | 51 | 1 | 42.2% | 96.4% | 17.7% | 34.6% | 91.7% |
| 0.99569429 | 27 | 12 | 50 | 1 | 43.3% | 96.4% | 19.4% | 35.1% | 92.3% |
| 0.9973545 | 27 | 13 | 49 | 1 | 44.4% | 96.4% | 21.0% | 35.5% | 92.9% |
| 0.99817784 | 27 | 14 | 48 | 1 | 45.6% | 96.4% | 22.6% | 36.0% | 93.3% |
| 1.00100908 | 27 | 15 | 47 | 1 | 46.7% | 96.4% | 24.2% | 36.5% | 93.8% |
| 1.00814111 | 27 | 16 | 46 | 1 | 47.8% | 96.4% | 25.8% | 37.0% | 94.1% |
| 1.01567398 | 27 | 17 | 45 | 1 | 48.9% | 96.4% | 27.4% | 37.5% | 94.4% |
| 1.01886792 | 27 | 18 | 44 | 1 | 50.0% | 96.4% | 29.0% | 38.0% | 94.7% |
| 1.01956703 | 27 | 19 | 43 | 1 | 51.1% | 96.4% | 30.6% | 38.6% | 95.0% |
| 1.05365355 | 27 | 20 | 42 | 1 | 52.2% | 96.4% | 32.3% | 39.1% | 95.2% |
| 1.05857988 | 27 | 21 | 41 | 1 | 53.3% | 96.4% | 33.9% | 39.7% | 95.5% |
| 1.07121484 | 27 | 22 | 40 | 1 | 54.4% | 96.4% | 35.5% | 40.3% | 95.7% |
| 1.10904007 | 27 | 23 | 39 | 1 | 55.6% | 96.4% | 37.1% | 40.9% | 95.8% |
| 1.11515152 | 27 | 24 | 38 | 1 | 56.7% | 96.4% | 38.7% | 41.5% | 96.0% |
| 1.12125639 | 27 | 25 | 37 | 1 | 57.8% | 96.4% | 40.3% | 42.2% | 96.2% |
| 1.12868217 | 27 | 26 | 36 | 1 | 58.9% | 96.4% | 41.9% | 42.9% | 96.3% |
| 1.15707621 | 27 | 27 | 35 | 1 | 60.0% | 96.4% | 43.5% | 43.5% | 96.4% |
| 1.17888563 | 27 | 28 | 34 | 1 | 61.1% | 96.4% | 45.2% | 44.3% | 96.6% |
| 1.18265541 | 27 | 29 | 33 | 1 | 62.2% | 96.4% | 46.8% | 45.0% | 96.7% |
| 1.18685955 | 27 | 30 | 32 | 1 | 63.3% | 96.4% | 48.4% | 45.8% | 96.8% |
| 1.19529719 | 27 | 31 | 31 | 1 | 64.4% | 96.4% | 50.0% | 46.6% | 96.9% |
| 1.21535064 | 27 | 32 | 30 | 1 | 65.6% | 96.4% | 51.6% | 47.4% | 97.0% |
| 1.2185008 | 27 | 33 | 29 | 1 | 66.7% | 96.4% | 53.2% | 48.2% | 97.1% |
| 1.22700297 | 27 | 34 | 28 | 1 | 67.8% | 96.4% | 54.8% | 49.1% | 97.1% |
| 1.46973094 | 26 | 34 | 28 | 2 | 66.7% | 92.9% | 54.8% | 48.1% | 94.4% |
| 1.48845382 | 26 | 35 | 27 | 2 | 67.8% | 92.9% | 56.5% | 49.1% | 94.6% |
| 1.49510033 | 26 | 36 | 26 | 2 | 68.9% | 92.9% | 58.1% | 50.0% | 94.7% |
| 1.516 | 25 | 36 | 26 | 3 | 67.8% | 89.3% | 58.1% | 49.0% | 92.3% |
| 1.51620029 | 25 | 37 | 25 | 3 | 68.9% | 89.3% | 59.7% | 50.0% | 92.5% |
| 1.54411765 | 24 | 37 | 25 | 4 | 67.8% | 85.7% | 59.7% | 49.0% | 90.2% |
| 1.56622024 | 23 | 37 | 25 | 5 | 66.7% | 82.1% | 59.7% | 47.9% | 88.1% |
| 1.56728972 | 23 | 38 | 24 | 5 | 67.8% | 82.1% | 61.3% | 48.9% | 88.4% |
| 1.56864654 | 22 | 38 | 24 | 6 | 66.7% | 78.6% | 61.3% | 47.8% | 86.4% |
| 1.59550562 | 21 | 38 | 24 | 7 | 65.6% | 75.0% | 61.3% | 46.7% | 84.4% |
| 1.63853503 | 21 | 39 | 23 | 7 | 66.7% | 75.0% | 62.9% | 47.7% | 84.8% |
| 1.68765957 | 20 | 39 | 23 | 8 | 65.6% | 71.4% | 62.9% | 46.5% | 83.0% |
| 1.69539615 | 20 | 40 | 22 | 8 | 66.7% | 71.4% | 64.5% | 47.6% | 83.3% |
| 1.71251499 | 20 | 41 | 21 | 8 | 67.8% | 71.4% | 66.1% | 48.8% | 83.7% |
| 1.72759857 | 20 | 42 | 20 | 8 | 68.9% | 71.4% | 67.7% | 50.0% | 84.0% |
| 1.77482447 | 20 | 43 | 19 | 8 | 70.0% | 71.4% | 69.4% | 51.3% | 84.3% |
| 1.83120205 | 20 | 44 | 18 | 8 | 71.1% | 71.4% | 71.0% | 52.6% | 84.6% |
| 1.85518814 | 20 | 45 | 17 | 8 | 72.2% | 71.4% | 72.6% | 54.1% | 84.9% |
| 1.85565419 | 19 | 45 | 17 | 9 | 71.1% | 67.9% | 72.6% | 52.8% | 83.3% |
| 1.86544462 | 18 | 45 | 17 | 10 | 70.0% | 64.3% | 72.6% | 51.4% | 81.8% |
| 1.87107172 | 17 | 45 | 17 | 11 | 68.9% | 60.7% | 72.6% | 50.0% | 80.4% |
| 1.87829111 | 17 | 46 | 16 | 11 | 70.0% | 60.7% | 74.2% | 51.5% | 80.7% |
| 1.94043887 | 16 | 46 | 16 | 12 | 68.9% | 57.1% | 74.2% | 50.0% | 79.3% |
| 1.95376791 | 16 | 47 | 15 | 12 | 70.0% | 57.1% | 75.8% | 51.6% | 79.7% |
| 1.96314908 | 15 | 47 | 15 | 13 | 68.9% | 53.6% | 75.8% | 50.0% | 78.3% |
| 2.07794944 | 15 | 48 | 14 | 13 | 70.0% | 53.6% | 77.4% | 51.7% | 78.7% |
| 2.08273749 | 15 | 49 | 13 | 13 | 71.1% | 53.6% | 79.0% | 53.6% | 79.0% |
| 2.10956175 | 15 | 50 | 12 | 13 | 72.2% | 53.6% | 80.6% | 55.6% | 79.4% |
| 2.13066826 | 14 | 50 | 12 | 14 | 71.1% | 50.0% | 80.6% | 53.8% | 78.1% |
| 2.13358779 | 14 | 51 | 11 | 14 | 72.2% | 50.0% | 82.3% | 56.0% | 78.5% |
| 2.18458781 | 13 | 51 | 11 | 15 | 71.1% | 46.4% | 82.3% | 54.2% | 77.3% |
| 2.18546196 | 13 | 52 | 10 | 15 | 72.2% | 46.4% | 83.9% | 56.5% | 77.6% |
| 2.25089286 | 13 | 53 | 9 | 15 | 73.3% | 46.4% | 85.5% | 59.1% | 77.9% |
| 2.27263875 | 12 | 53 | 9 | 16 | 72.2% | 42.9% | 85.5% | 57.1% | 76.8% |
| 2.32739212 | 12 | 54 | 8 | 16 | 73.3% | 42.9% | 87.1% | 60.0% | 77.1% |
| 2.38488576 | 11 | 54 | 8 | 17 | 72.2% | 39.3% | 87.1% | 57.9% | 76.1% |
| 2.40294985 | 10 | 54 | 8 | 18 | 71.1% | 35.7% | 87.1% | 55.6% | 75.0% |
| 2.41672727 | 9 | 54 | 8 | 19 | 70.0% | 32.1% | 87.1% | 52.9% | 74.0% |
| 2.44410256 | 8 | 54 | 8 | 20 | 68.9% | 28.6% | 87.1% | 50.0% | 73.0% |
| 2.44752093 | 7 | 54 | 8 | 21 | 67.8% | 25.0% | 87.1% | 46.7% | 72.0% |
| 2.44758735 | 6 | 54 | 8 | 22 | 66.7% | 21.4% | 87.1% | 42.9% | 71.1% |
| 2.45207101 | 6 | 55 | 7 | 22 | 67.8% | 21.4% | 88.7% | 46.2% | 71.4% |
| 2.48453608 | 6 | 56 | 6 | 22 | 68.9% | 21.4% | 90.3% | 50.0% | 71.8% |
| 2.6496199 | 5 | 56 | 6 | 23 | 67.8% | 17.9% | 90.3% | 45.5% | 70.9% |
| 2.740553 | 5 | 57 | 5 | 23 | 68.9% | 17.9% | 91.9% | 50.0% | 71.3% |
| 2.81826321 | 4 | 57 | 5 | 24 | 67.8% | 14.3% | 91.9% | 44.4% | 70.4% |
| 2.82761194 | 3 | 57 | 5 | 25 | 66.7% | 10.7% | 91.9% | 37.5% | 69.5% |
| 2.87229862 | 2 | 57 | 5 | 26 | 65.6% | 7.1% | 91.9% | 28.6% | 68.7% |
| 2.89291883 | 2 | 58 | 4 | 26 | 66.7% | 7.1% | 93.5% | 33.3% | 69.0% |
| 3.28316611 | 2 | 59 | 3 | 26 | 67.8% | 7.1% | 95.2% | 40.0% | 69.4% |
| 3.28534031 | 1 | 59 | 3 | 27 | 66.7% | 3.6% | 95.2% | 25.0% | 68.6% |
| 3.30438066 | 1 | 60 | 2 | 27 | 67.8% | 3.6% | 96.8% | 33.3% | 69.0% |
| 3.49430797 | 0 | 60 | 2 | 28 | 66.7% | 0.0% | 96.8% | 0.0% | 68.2% |
| 3.57142857 | 0 | 61 | 1 | 28 | 67.8% | 0.0% | 98.4% | 0.0% | 68.5% |
| 3.68801653 | 0 | 62 | 0 | 28 | 68.9% | 0.0% | 100.0% | NA | 68.9% |

## HR+HER2+

**MrE_Size: PostMR early phase (cm)**

| MrE_Size | Correct | | Incorrect | | Percentages | | | | |
|---|---|---|---|---|---|---|---|---|---|
| | Event | Non-Event | Event | Non-Event | Correct | Sensitivity | Specificity | PPV | NPV |
| 0 | 51 | 20 | 22 | 10 | 68.9% | 83.6% | 47.6% | 69.9% | 66.7% |
| 0.2 | 47 | 23 | 19 | 14 | 68.0% | 77.0% | 54.8% | 71.2% | 62.2% |
| 0.3 | 44 | 27 | 15 | 17 | 68.9% | 72.1% | 64.3% | 74.6% | 61.4% |
| 0.4 | 41 | 28 | 14 | 20 | 67.0% | 67.2% | 66.7% | 74.5% | 58.3% |

| | | | | | | | | | |
|---|---|---|---|---|---|---|---|---|---|
| 0.5 | 41 | 29 | 13 | 20 | 68.0% | 67.2% | 69.0% | 75.9% | 59.2% |
| 0.6 | 38 | 30 | 12 | 23 | 66.0% | 62.3% | 71.4% | 76.0% | 56.6% |
| 0.7 | 37 | 32 | 10 | 24 | 67.0% | 60.7% | 76.2% | 78.7% | 57.1% |
| 0.8 | 33 | 34 | 8 | 28 | 65.0% | 54.1% | 81.0% | 80.5% | 54.8% |
| 0.9 | 33 | 36 | 6 | 28 | 67.0% | 54.1% | 85.7% | 84.6% | 56.3% |
| 1 | 30 | 38 | 4 | 31 | 66.0% | 49.2% | 90.5% | 88.2% | 55.1% |
| 1.1 | 26 | 38 | 4 | 35 | 62.1% | 42.6% | 90.5% | 86.7% | 52.1% |
| 1.2 | 25 | 38 | 4 | 36 | 61.2% | 41.0% | 90.5% | 86.2% | 51.4% |
| 1.3 | 20 | 38 | 4 | 41 | 56.3% | 32.8% | 90.5% | 83.3% | 48.1% |
| 1.4 | 20 | 39 | 3 | 41 | 57.3% | 32.8% | 92.9% | 87.0% | 48.8% |
| 1.5 | 19 | 39 | 3 | 42 | 56.3% | 31.1% | 92.9% | 86.4% | 48.1% |
| 1.6 | 16 | 39 | 3 | 45 | 53.4% | 26.2% | 92.9% | 84.2% | 46.4% |
| 1.7 | 15 | 40 | 2 | 46 | 53.4% | 24.6% | 95.2% | 88.2% | 46.5% |
| 1.8 | 14 | 40 | 2 | 47 | 52.4% | 23.0% | 95.2% | 87.5% | 46.0% |
| 1.9 | 12 | 40 | 2 | 49 | 50.5% | 19.7% | 95.2% | 85.7% | 44.9% |
| 2 | 11 | 40 | 2 | 50 | 49.5% | 18.0% | 95.2% | 84.6% | 44.4% |
| 2.1 | 10 | 40 | 2 | 51 | 48.5% | 16.4% | 95.2% | 83.3% | 44.0% |
| 2.3 | 8 | 40 | 2 | 53 | 46.6% | 13.1% | 95.2% | 80.0% | 43.0% |
| 2.4 | 6 | 40 | 2 | 55 | 44.7% | 9.8% | 95.2% | 75.0% | 42.1% |
| 2.5 | 5 | 40 | 2 | 56 | 43.7% | 8.2% | 95.2% | 71.4% | 41.7% |
| 2.8 | 4 | 40 | 2 | 57 | 42.7% | 6.6% | 95.2% | 66.7% | 41.2% |
| 3.5 | 3 | 40 | 2 | 58 | 41.7% | 4.9% | 95.2% | 60.0% | 40.8% |
| 3.9 | 2 | 40 | 2 | 59 | 40.8% | 3.3% | 95.2% | 50.0% | 40.4% |
| 5 | 2 | 41 | 1 | 59 | 41.7% | 3.3% | 97.6% | 66.7% | 41.0% |
| 5.5 | 0 | 41 | 1 | 61 | 39.8% | 0.0% | 97.6% | 0.0% | 40.2% |
| 6 | 0 | 42 | 0 | 61 | 40.8% | 0.0% | 100.0% | NA | 40.8% |

**MrL_Size: PostMR late phase (cm)**

| MrL_Size | Correct | | Incorrect | | Percentages | | | | |
|---|---|---|---|---|---|---|---|---|---|
| | Event | Non-Event | Event | Non-Event | Correct | Sensitivity | Specificity | PPV | NPV |
| 0 | 54 | 14 | 28 | 7 | 66.0% | 88.5% | 33.3% | 65.9% | 66.7% |
| 0.2 | 53 | 15 | 27 | 8 | 66.0% | 86.9% | 35.7% | 66.3% | 65.2% |
| 0.3 | 47 | 17 | 25 | 14 | 62.1% | 77.0% | 40.5% | 65.3% | 54.8% |
| 0.4 | 44 | 20 | 22 | 17 | 62.1% | 72.1% | 47.6% | 66.7% | 54.1% |
| 0.6 | 41 | 22 | 20 | 20 | 61.2% | 67.2% | 52.4% | 67.2% | 52.4% |
| 0.7 | 40 | 23 | 19 | 21 | 61.2% | 65.6% | 54.8% | 67.8% | 52.3% |
| 0.8 | 36 | 24 | 18 | 25 | 58.3% | 59.0% | 57.1% | 66.7% | 49.0% |
| 1 | 32 | 30 | 12 | 29 | 60.2% | 52.5% | 71.4% | 72.7% | 50.8% |
| 1.1 | 28 | 30 | 12 | 33 | 56.3% | 45.9% | 71.4% | 70.0% | 47.6% |
| 1.2 | 27 | 34 | 8 | 34 | 59.2% | 44.3% | 81.0% | 77.1% | 50.0% |
| 1.3 | 22 | 34 | 8 | 39 | 54.4% | 36.1% | 81.0% | 73.3% | 46.6% |
| 1.5 | 21 | 35 | 7 | 40 | 54.4% | 34.4% | 83.3% | 75.0% | 46.7% |
| 1.6 | 19 | 36 | 6 | 42 | 53.4% | 31.1% | 85.7% | 76.0% | 46.2% |
| 1.8 | 17 | 36 | 6 | 44 | 51.5% | 27.9% | 85.7% | 73.9% | 45.0% |
| 1.9 | 15 | 36 | 6 | 46 | 49.5% | 24.6% | 85.7% | 71.4% | 43.9% |
| 2 | 13 | 38 | 4 | 48 | 49.5% | 21.3% | 90.5% | 76.5% | 44.2% |
| 2.1 | 12 | 38 | 4 | 49 | 48.5% | 19.7% | 90.5% | 75.0% | 43.7% |
| 2.2 | 11 | 38 | 4 | 50 | 47.6% | 18.0% | 90.5% | 73.3% | 43.2% |
| 2.3 | 9 | 38 | 4 | 52 | 45.6% | 14.8% | 90.5% | 69.2% | 42.2% |
| 2.4 | 7 | 38 | 4 | 54 | 43.7% | 11.5% | 90.5% | 63.6% | 41.3% |
| 2.5 | 6 | 39 | 3 | 55 | 43.7% | 9.8% | 92.9% | 66.7% | 41.5% |
| 2.8 | 5 | 39 | 3 | 56 | 42.7% | 8.2% | 92.9% | 62.5% | 41.1% |
| 3.5 | 4 | 39 | 3 | 57 | 41.7% | 6.6% | 92.9% | 57.1% | 40.6% |
| 3.8 | 3 | 39 | 3 | 58 | 40.8% | 4.9% | 92.9% | 50.0% | 40.2% |
| 3.9 | 2 | 39 | 3 | 59 | 39.8% | 3.3% | 92.9% | 40.0% | 39.8% |
| 5 | 2 | 40 | 2 | 59 | 40.8% | 3.3% | 95.2% | 50.0% | 40.4% |
| 5.5 | 0 | 40 | 2 | 61 | 38.8% | 0.0% | 95.2% | 0.0% | 39.6% |
| 6 | 0 | 41 | 1 | 61 | 39.8% | 0.0% | 97.6% | 0.0% | 40.2% |
| 11 | 0 | 42 | 0 | 61 | 40.8% | 0.0% | 100.0% | NA | 40.8% |

**MrSerE: Lesion-to-background SER (early)**

| MrSerE | Correct | | Incorrect | | Percentages | | | | |
|---|---|---|---|---|---|---|---|---|---|
| | Event | Non-Event | Event | Non-Event | Correct | Sensitivity | Specificity | PPV | NPV |
| 0.34389562 | 61 | 1 | 41 | 0 | 60.2% | 100.0% | 2.4% | 59.8% | 100.0% |
| 0.55216693 | 60 | 1 | 41 | 1 | 59.2% | 98.4% | 2.4% | 59.4% | 50.0% |
| 0.74828375 | 60 | 2 | 40 | 1 | 60.2% | 98.4% | 4.8% | 60.0% | 66.7% |
| 0.7626539 | 59 | 2 | 40 | 2 | 59.2% | 96.7% | 4.8% | 59.6% | 50.0% |
| 0.80233528 | 59 | 3 | 39 | 2 | 60.2% | 96.7% | 7.1% | 60.2% | 60.0% |
| 0.86610169 | 59 | 4 | 38 | 2 | 61.2% | 96.7% | 9.5% | 60.8% | 66.7% |
| 0.87216495 | 59 | 5 | 37 | 2 | 62.1% | 96.7% | 11.9% | 61.5% | 71.4% |
| 0.89464286 | 59 | 6 | 36 | 2 | 63.1% | 96.7% | 14.3% | 62.1% | 75.0% |
| 0.91417278 | 59 | 7 | 35 | 2 | 64.1% | 96.7% | 16.7% | 62.8% | 77.8% |
| 0.91809524 | 59 | 8 | 34 | 2 | 65.0% | 96.7% | 19.0% | 63.4% | 80.0% |
| 0.94228356 | 59 | 9 | 33 | 2 | 66.0% | 96.7% | 21.4% | 64.1% | 81.8% |
| 0.94619883 | 59 | 10 | 32 | 2 | 67.0% | 96.7% | 23.8% | 64.8% | 83.3% |
| 0.95106589 | 59 | 11 | 31 | 2 | 68.0% | 96.7% | 26.2% | 65.6% | 84.6% |
| 0.97624818 | 59 | 12 | 30 | 2 | 68.9% | 96.7% | 28.6% | 66.3% | 85.7% |
| 0.98041237 | 58 | 12 | 30 | 3 | 68.0% | 95.1% | 28.6% | 65.9% | 80.0% |
| 0.9826969 | 57 | 12 | 30 | 4 | 67.0% | 93.4% | 28.6% | 65.5% | 75.0% |
| 0.98329854 | 57 | 13 | 29 | 4 | 68.0% | 93.4% | 31.0% | 66.3% | 76.5% |
| 0.99524376 | 57 | 14 | 28 | 4 | 68.9% | 93.4% | 33.3% | 67.1% | 77.8% |
| 1.00621891 | 56 | 14 | 28 | 5 | 68.0% | 91.8% | 33.3% | 66.7% | 73.7% |
| 1.01033386 | 56 | 15 | 27 | 5 | 68.9% | 91.8% | 35.7% | 67.5% | 75.0% |
| 1.01699926 | 56 | 16 | 26 | 5 | 69.9% | 91.8% | 38.1% | 68.3% | 76.2% |
| 1.02889667 | 55 | 16 | 26 | 6 | 68.9% | 90.2% | 38.1% | 67.9% | 72.7% |
| 1.04904632 | 55 | 17 | 25 | 6 | 69.9% | 90.2% | 40.5% | 68.8% | 73.9% |
| 1.05865922 | 55 | 18 | 24 | 6 | 70.9% | 90.2% | 42.9% | 69.6% | 75.0% |
| 1.09379128 | 55 | 19 | 23 | 6 | 71.8% | 90.2% | 45.2% | 70.5% | 76.0% |
| 1.11808118 | 54 | 19 | 23 | 7 | 70.9% | 88.5% | 45.2% | 70.1% | 73.1% |
| 1.14941691 | 53 | 19 | 23 | 8 | 69.9% | 86.9% | 45.2% | 69.7% | 70.4% |
| 1.15785609 | 52 | 19 | 23 | 9 | 68.9% | 85.2% | 45.2% | 69.3% | 67.9% |
| 1.198 | 52 | 20 | 22 | 9 | 69.9% | 85.2% | 47.6% | 70.3% | 69.0% |
| 1.20748299 | 52 | 21 | 21 | 9 | 70.9% | 85.2% | 50.0% | 71.2% | 70.0% |
| 1.2132964 | 51 | 21 | 21 | 10 | 69.9% | 83.6% | 50.0% | 70.8% | 67.7% |
| 1.22439024 | 50 | 21 | 21 | 11 | 68.9% | 82.0% | 50.0% | 70.4% | 65.6% |
| 1.24113475 | 50 | 22 | 20 | 11 | 69.9% | 82.0% | 52.4% | 71.4% | 66.7% |
| 1.25651042 | 49 | 22 | 20 | 12 | 68.9% | 80.3% | 52.4% | 71.0% | 64.7% |
| 1.25929204 | 49 | 23 | 19 | 12 | 69.9% | 80.3% | 54.8% | 72.1% | 65.7% |
| 1.27564392 | 49 | 24 | 18 | 12 | 70.9% | 80.3% | 57.1% | 73.1% | 66.7% |
| 1.34486546 | 49 | 25 | 17 | 12 | 71.8% | 80.3% | 59.5% | 74.2% | 67.6% |
| 1.37180185 | 48 | 25 | 17 | 13 | 70.9% | 78.7% | 59.5% | 73.8% | 65.8% |
| 1.38544799 | 48 | 26 | 16 | 13 | 71.8% | 78.7% | 61.9% | 75.0% | 66.7% |
| 1.39806678 | 48 | 27 | 15 | 13 | 72.8% | 78.7% | 64.3% | 76.2% | 67.5% |

| | | | | | | | | | |
|---|---|---|---|---|---|---|---|---|---|
| 1.41266376 | 47 | 27 | 15 | 14 | 71.8% | 77.0% | 64.3% | 75.8% | 65.9% |
| 1.41919192 | 47 | 28 | 14 | 14 | 72.8% | 77.0% | 66.7% | 77.0% | 66.7% |
| 1.50304878 | 46 | 28 | 14 | 15 | 71.8% | 75.4% | 66.7% | 76.7% | 65.1% |
| 1.53409091 | 45 | 28 | 14 | 16 | 70.9% | 73.8% | 66.7% | 76.3% | 63.6% |
| 1.54002463 | 45 | 29 | 13 | 16 | 71.8% | 73.8% | 69.0% | 77.6% | 64.4% |
| 1.57950907 | 44 | 29 | 13 | 17 | 70.9% | 72.1% | 69.0% | 77.2% | 63.0% |
| 1.59695817 | 43 | 29 | 13 | 18 | 69.9% | 70.5% | 69.0% | 76.8% | 61.7% |
| 1.60319767 | 43 | 30 | 12 | 18 | 70.9% | 70.5% | 71.4% | 78.2% | 62.5% |
| 1.60883036 | 42 | 30 | 12 | 19 | 69.9% | 68.9% | 71.4% | 77.8% | 61.2% |
| 1.61132966 | 41 | 30 | 12 | 20 | 68.9% | 67.2% | 71.4% | 77.4% | 60.0% |
| 1.61758691 | 40 | 30 | 12 | 21 | 68.0% | 65.6% | 71.4% | 76.9% | 58.8% |
| 1.62225587 | 40 | 31 | 11 | 21 | 68.9% | 65.6% | 73.8% | 78.4% | 59.6% |
| 1.65443629 | 40 | 32 | 10 | 21 | 69.9% | 65.6% | 76.2% | 80.0% | 60.4% |
| 1.66248257 | 39 | 32 | 10 | 22 | 68.9% | 63.9% | 76.2% | 79.6% | 59.3% |
| 1.70845481 | 38 | 32 | 10 | 23 | 68.0% | 62.3% | 76.2% | 79.2% | 58.2% |
| 1.71078431 | 37 | 32 | 10 | 24 | 67.0% | 60.7% | 76.2% | 78.7% | 57.1% |
| 1.72467903 | 36 | 32 | 10 | 25 | 66.0% | 59.0% | 76.2% | 78.3% | 56.1% |
| 1.74256506 | 36 | 33 | 9 | 25 | 67.0% | 59.0% | 78.6% | 80.0% | 56.9% |
| 1.74269341 | 35 | 33 | 9 | 26 | 66.0% | 57.4% | 78.6% | 79.5% | 55.9% |
| 1.77043765 | 35 | 34 | 8 | 26 | 67.0% | 57.4% | 81.0% | 81.4% | 56.7% |
| 1.81781377 | 34 | 34 | 8 | 27 | 66.0% | 55.7% | 81.0% | 81.0% | 55.7% |
| 1.83435048 | 33 | 34 | 8 | 28 | 65.0% | 54.1% | 81.0% | 80.5% | 54.8% |
| 1.85701107 | 32 | 34 | 8 | 29 | 64.1% | 52.5% | 81.0% | 80.0% | 54.0% |
| 1.86839968 | 31 | 34 | 8 | 30 | 63.1% | 50.8% | 81.0% | 79.5% | 53.1% |
| 1.90133333 | 31 | 35 | 7 | 30 | 64.1% | 50.8% | 83.3% | 81.6% | 53.8% |
| 1.91928975 | 30 | 35 | 7 | 31 | 63.1% | 49.2% | 83.3% | 81.1% | 53.0% |
| 1.93886861 | 29 | 35 | 7 | 32 | 62.1% | 47.5% | 83.3% | 80.6% | 52.2% |
| 1.93910256 | 28 | 35 | 7 | 33 | 61.2% | 45.9% | 83.3% | 80.0% | 51.5% |
| 2.01179245 | 27 | 35 | 7 | 34 | 60.2% | 44.3% | 83.3% | 79.4% | 50.7% |
| 2.0245614 | 26 | 35 | 7 | 35 | 59.2% | 42.6% | 83.3% | 78.8% | 50.0% |
| 2.03221188 | 25 | 35 | 7 | 36 | 58.3% | 41.0% | 83.3% | 78.1% | 49.3% |
| 2.03580563 | 24 | 35 | 7 | 37 | 57.3% | 39.3% | 83.3% | 77.4% | 48.6% |
| 2.10877404 | 23 | 35 | 7 | 38 | 56.3% | 37.7% | 83.3% | 76.7% | 47.9% |
| 2.15561759 | 23 | 36 | 6 | 38 | 57.3% | 37.7% | 85.7% | 79.3% | 48.6% |
| 2.16647264 | 22 | 36 | 6 | 39 | 56.3% | 36.1% | 85.7% | 78.6% | 48.0% |
| 2.18330733 | 21 | 36 | 6 | 40 | 55.3% | 34.4% | 85.7% | 77.8% | 47.4% |
| 2.23047977 | 20 | 36 | 6 | 41 | 54.4% | 32.8% | 85.7% | 76.9% | 46.8% |
| 2.27232143 | 19 | 36 | 6 | 42 | 53.4% | 31.1% | 85.7% | 76.0% | 46.2% |
| 2.27944372 | 18 | 36 | 6 | 43 | 52.4% | 29.5% | 85.7% | 75.0% | 45.6% |
| 2.28733766 | 18 | 37 | 5 | 43 | 53.4% | 29.5% | 88.1% | 78.3% | 46.3% |
| 2.29125413 | 18 | 38 | 4 | 43 | 54.4% | 29.5% | 90.5% | 81.8% | 46.9% |
| 2.31786543 | 18 | 39 | 3 | 43 | 55.3% | 29.5% | 92.9% | 85.7% | 47.6% |
| 2.34519573 | 18 | 40 | 2 | 43 | 56.3% | 29.5% | 95.2% | 90.0% | 48.2% |
| 2.37343533 | 17 | 40 | 2 | 44 | 55.3% | 27.9% | 95.2% | 89.5% | 47.6% |
| 2.46930423 | 16 | 40 | 2 | 45 | 54.4% | 26.2% | 95.2% | 88.9% | 47.1% |
| 2.47402597 | 15 | 40 | 2 | 46 | 53.4% | 24.6% | 95.2% | 88.2% | 46.5% |
| 2.56454491 | 14 | 40 | 2 | 47 | 52.4% | 23.0% | 95.2% | 87.5% | 46.0% |
| 2.62469237 | 13 | 40 | 2 | 48 | 51.5% | 21.3% | 95.2% | 86.7% | 45.5% |
| 2.64260563 | 12 | 40 | 2 | 49 | 50.5% | 19.7% | 95.2% | 85.7% | 44.9% |
| 2.72140575 | 11 | 40 | 2 | 50 | 49.5% | 18.0% | 95.2% | 84.6% | 44.4% |
| 2.7902439 | 10 | 40 | 2 | 51 | 48.5% | 16.4% | 95.2% | 83.3% | 44.0% |
| 2.79057592 | 9 | 40 | 2 | 52 | 47.6% | 14.8% | 95.2% | 81.8% | 43.5% |
| 2.80732314 | 8 | 40 | 2 | 53 | 46.6% | 13.1% | 95.2% | 80.0% | 43.0% |
| 3.07796917 | 7 | 40 | 2 | 54 | 45.6% | 11.5% | 95.2% | 77.8% | 42.6% |
| 3.08176944 | 6 | 40 | 2 | 55 | 44.7% | 9.8% | 95.2% | 75.0% | 42.1% |
| 3.13043478 | 5 | 40 | 2 | 56 | 43.7% | 8.2% | 95.2% | 71.4% | 41.7% |
| 3.19496855 | 5 | 41 | 1 | 56 | 44.7% | 8.2% | 97.6% | 83.3% | 42.3% |
| 3.39520958 | 4 | 41 | 1 | 57 | 43.7% | 6.6% | 97.6% | 80.0% | 41.8% |
| 3.5183763 | 3 | 41 | 1 | 58 | 42.7% | 4.9% | 97.6% | 75.0% | 41.4% |
| 3.59411362 | 2 | 41 | 1 | 59 | 41.7% | 3.3% | 97.6% | 66.7% | 41.0% |
| 3.60223368 | 2 | 42 | 0 | 59 | 42.7% | 3.3% | 100.0% | 100.0% | 41.6% |
| 3.78670635 | 1 | 42 | 0 | 60 | 41.7% | 1.6% | 100.0% | 100.0% | 41.2% |
| 4.10933941 | 0 | 42 | 0 | 61 | 40.8% | 0.0% | 100.0% | NA | 40.8% |

**MrSerL: Lesion-to-background SER (late)**

| MrSerL | Correct | | Incorrect | | Percentages | | | | |
|---|---|---|---|---|---|---|---|---|---|
| | Event | Non-Event | Event | Non-Event | Correct | Sensitivity | Specificity | PPV | NPV |
| 0.40951572 | 61 | 1 | 41 | 0 | 60.2% | 100.0% | 2.4% | 59.8% | 100.0% |
| 0.73084112 | 60 | 1 | 41 | 1 | 59.2% | 98.4% | 2.4% | 59.4% | 50.0% |
| 0.81752874 | 59 | 1 | 41 | 2 | 58.3% | 96.7% | 2.4% | 59.0% | 33.3% |
| 0.91709464 | 58 | 1 | 41 | 3 | 57.3% | 95.1% | 2.4% | 58.6% | 25.0% |
| 0.93632959 | 58 | 2 | 40 | 3 | 58.3% | 95.1% | 4.8% | 59.2% | 40.0% |
| 0.96314103 | 57 | 2 | 40 | 4 | 57.3% | 93.4% | 4.8% | 58.8% | 33.3% |
| 0.96418129 | 57 | 3 | 39 | 4 | 58.3% | 93.4% | 7.1% | 59.4% | 42.9% |
| 0.96767677 | 56 | 3 | 39 | 5 | 57.3% | 91.8% | 7.1% | 58.9% | 37.5% |
| 0.984375 | 56 | 4 | 38 | 5 | 58.3% | 91.8% | 9.5% | 59.6% | 44.4% |
| 0.98575499 | 56 | 5 | 37 | 5 | 59.2% | 91.8% | 11.9% | 60.2% | 50.0% |
| 0.99127676 | 56 | 6 | 36 | 5 | 60.2% | 91.8% | 14.3% | 60.9% | 54.5% |
| 0.99412393 | 56 | 7 | 35 | 5 | 61.2% | 91.8% | 16.7% | 61.5% | 58.3% |
| 1.00460035 | 55 | 7 | 35 | 6 | 60.2% | 90.2% | 16.7% | 61.1% | 53.8% |
| 1.00571429 | 55 | 8 | 34 | 6 | 61.2% | 90.2% | 19.0% | 61.8% | 57.1% |
| 1.00578947 | 55 | 9 | 33 | 6 | 62.1% | 90.2% | 21.4% | 62.5% | 60.0% |
| 1.00909091 | 55 | 10 | 32 | 6 | 63.1% | 90.2% | 23.8% | 63.2% | 62.5% |
| 1.01393095 | 54 | 10 | 32 | 7 | 62.1% | 88.5% | 23.8% | 62.8% | 58.8% |
| 1.04171882 | 54 | 11 | 31 | 7 | 63.1% | 88.5% | 26.2% | 63.5% | 61.1% |
| 1.09298999 | 54 | 12 | 30 | 7 | 64.1% | 88.5% | 28.6% | 64.3% | 63.2% |
| 1.11770335 | 53 | 12 | 30 | 8 | 63.1% | 86.9% | 28.6% | 63.9% | 60.0% |
| 1.12869565 | 53 | 13 | 29 | 8 | 64.1% | 86.9% | 31.0% | 64.6% | 61.9% |
| 1.13383459 | 53 | 14 | 28 | 8 | 65.0% | 86.9% | 33.3% | 65.4% | 63.6% |
| 1.13559322 | 52 | 14 | 28 | 9 | 64.1% | 85.2% | 33.3% | 65.0% | 60.9% |
| 1.17308607 | 52 | 15 | 27 | 9 | 65.0% | 85.2% | 35.7% | 65.8% | 62.5% |
| 1.21466131 | 52 | 16 | 26 | 9 | 66.0% | 85.2% | 38.1% | 66.7% | 64.0% |
| 1.2352071 | 52 | 17 | 25 | 9 | 67.0% | 85.2% | 40.5% | 67.5% | 65.4% |
| 1.24067389 | 52 | 18 | 24 | 9 | 68.0% | 85.2% | 42.9% | 68.4% | 66.7% |
| 1.27035831 | 51 | 18 | 24 | 10 | 67.0% | 83.6% | 42.9% | 68.0% | 64.3% |
| 1.2932579 | 51 | 19 | 23 | 10 | 68.0% | 83.6% | 45.2% | 68.9% | 65.5% |
| 1.30973451 | 51 | 20 | 22 | 10 | 68.9% | 83.6% | 47.6% | 69.9% | 66.7% |
| 1.31068648 | 51 | 21 | 21 | 10 | 69.9% | 83.6% | 50.0% | 70.8% | 67.7% |
| 1.32523468 | 50 | 21 | 21 | 11 | 68.9% | 82.0% | 50.0% | 70.4% | 65.6% |
| 1.37605178 | 50 | 22 | 20 | 11 | 69.9% | 82.0% | 52.4% | 71.4% | 66.7% |
| 1.39803171 | 50 | 23 | 19 | 11 | 70.9% | 82.0% | 54.8% | 72.5% | 67.6% |
| 1.40612648 | 50 | 24 | 18 | 11 | 71.8% | 82.0% | 57.1% | 73.5% | 68.6% |
| 1.42307692 | 50 | 25 | 17 | 11 | 72.8% | 82.0% | 59.5% | 74.6% | 69.4% |
| 1.42546584 | 50 | 26 | 16 | 11 | 73.8% | 82.0% | 61.9% | 75.8% | 70.3% |
| 1.44942592 | 50 | 27 | 15 | 11 | 74.8% | 82.0% | 64.3% | 76.9% | 71.1% |
| 1.51616162 | 49 | 27 | 15 | 12 | 73.8% | 80.3% | 64.3% | 76.6% | 69.2% |
| 1.51863354 | 48 | 27 | 15 | 13 | 72.8% | 78.7% | 64.3% | 76.2% | 67.5% |
| 1.53495935 | 47 | 27 | 15 | 14 | 71.8% | 77.0% | 64.3% | 75.8% | 65.9% |

| | | | | | | | | | |
|---|---|---|---|---|---|---|---|---|---|
| 1.54975845 | 46 | 27 | 15 | 15 | 70.9% | 75.4% | 64.3% | 75.4% | 64.3% |
| 1.57057869 | 45 | 27 | 15 | 16 | 69.9% | 73.8% | 64.3% | 75.0% | 62.8% |
| 1.59441805 | 45 | 28 | 14 | 16 | 70.9% | 73.8% | 66.7% | 76.3% | 63.6% |
| 1.60127389 | 44 | 28 | 14 | 17 | 69.9% | 72.1% | 66.7% | 75.9% | 62.2% |
| 1.61266748 | 43 | 28 | 14 | 18 | 68.9% | 70.5% | 66.7% | 75.4% | 60.9% |
| 1.62763466 | 43 | 29 | 13 | 18 | 69.9% | 70.5% | 69.0% | 76.8% | 61.7% |
| 1.66273932 | 42 | 29 | 13 | 19 | 68.9% | 68.9% | 69.0% | 76.4% | 60.4% |
| 1.66987741 | 41 | 29 | 13 | 20 | 68.0% | 67.2% | 69.0% | 75.9% | 59.2% |
| 1.67039764 | 40 | 29 | 13 | 21 | 67.0% | 65.6% | 69.0% | 75.5% | 58.0% |
| 1.67807263 | 39 | 29 | 13 | 22 | 66.0% | 63.9% | 69.0% | 75.0% | 56.9% |
| 1.69149498 | 38 | 29 | 13 | 23 | 65.0% | 62.3% | 69.0% | 74.5% | 55.8% |
| 1.69236209 | 38 | 30 | 12 | 23 | 66.0% | 62.3% | 71.4% | 76.0% | 56.6% |
| 1.70446233 | 38 | 31 | 11 | 23 | 67.0% | 62.3% | 73.8% | 77.6% | 57.4% |
| 1.7194781 | 38 | 32 | 10 | 23 | 68.0% | 62.3% | 76.2% | 79.2% | 58.2% |
| 1.73563218 | 37 | 32 | 10 | 24 | 67.0% | 60.7% | 76.2% | 78.7% | 57.1% |
| 1.73616734 | 36 | 32 | 10 | 25 | 66.0% | 59.0% | 76.2% | 78.3% | 56.1% |
| 1.74873354 | 35 | 32 | 10 | 26 | 65.0% | 57.4% | 76.2% | 77.8% | 55.2% |
| 1.77521614 | 35 | 33 | 9 | 26 | 66.0% | 57.4% | 78.6% | 79.5% | 55.9% |
| 1.8126195 | 35 | 34 | 8 | 26 | 67.0% | 57.4% | 81.0% | 81.4% | 56.7% |
| 1.81524725 | 34 | 34 | 8 | 27 | 66.0% | 55.7% | 81.0% | 81.0% | 55.7% |
| 1.82891832 | 33 | 34 | 8 | 28 | 65.0% | 54.1% | 81.0% | 80.5% | 54.8% |
| 1.83423913 | 32 | 34 | 8 | 29 | 64.1% | 52.5% | 81.0% | 80.0% | 54.0% |
| 1.86377001 | 31 | 34 | 8 | 30 | 63.1% | 50.8% | 81.0% | 79.5% | 53.1% |
| 1.8643617 | 31 | 35 | 7 | 30 | 64.1% | 50.8% | 83.3% | 81.6% | 53.8% |
| 1.86544046 | 30 | 35 | 7 | 31 | 63.1% | 49.2% | 83.3% | 81.1% | 53.0% |
| 1.86790698 | 30 | 36 | 6 | 31 | 64.1% | 49.2% | 85.7% | 83.3% | 53.7% |
| 1.92784314 | 30 | 37 | 5 | 31 | 65.0% | 49.2% | 88.1% | 85.7% | 54.4% |
| 1.93788438 | 29 | 37 | 5 | 32 | 64.1% | 47.5% | 88.1% | 85.3% | 53.6% |
| 1.99300699 | 28 | 37 | 5 | 33 | 63.1% | 45.9% | 88.1% | 84.8% | 52.9% |
| 1.9956976 | 28 | 38 | 4 | 33 | 64.1% | 45.9% | 90.5% | 87.5% | 53.5% |
| 2.01329535 | 27 | 38 | 4 | 34 | 63.1% | 44.3% | 90.5% | 87.1% | 52.8% |
| 2.03382664 | 26 | 38 | 4 | 35 | 62.1% | 42.6% | 90.5% | 86.7% | 52.1% |
| 2.04479167 | 25 | 38 | 4 | 36 | 61.2% | 41.0% | 90.5% | 86.2% | 51.4% |
| 2.06115108 | 24 | 38 | 4 | 37 | 60.2% | 39.3% | 90.5% | 85.7% | 50.7% |
| 2.06932271 | 23 | 38 | 4 | 38 | 59.2% | 37.7% | 90.5% | 85.2% | 50.0% |
| 2.07041276 | 22 | 38 | 4 | 39 | 58.3% | 36.1% | 90.5% | 84.6% | 49.4% |
| 2.10196906 | 21 | 38 | 4 | 40 | 57.3% | 34.4% | 90.5% | 84.0% | 48.7% |
| 2.11041504 | 20 | 38 | 4 | 41 | 56.3% | 32.8% | 90.5% | 83.3% | 48.1% |
| 2.11151737 | 19 | 38 | 4 | 42 | 55.3% | 31.1% | 90.5% | 82.6% | 47.5% |
| 2.11742424 | 18 | 38 | 4 | 43 | 54.4% | 29.5% | 90.5% | 81.8% | 46.9% |
| 2.12643678 | 17 | 38 | 4 | 44 | 53.4% | 27.9% | 90.5% | 81.0% | 46.3% |
| 2.13280866 | 16 | 38 | 4 | 45 | 52.4% | 26.2% | 90.5% | 80.0% | 45.8% |
| 2.14138817 | 16 | 39 | 3 | 45 | 53.4% | 26.2% | 92.9% | 84.2% | 46.4% |
| 2.21293574 | 15 | 39 | 3 | 46 | 52.4% | 24.6% | 92.9% | 83.3% | 45.9% |
| 2.26040268 | 14 | 39 | 3 | 47 | 51.5% | 23.0% | 92.9% | 82.4% | 45.3% |
| 2.31325301 | 13 | 39 | 3 | 48 | 50.5% | 21.3% | 92.9% | 81.3% | 44.8% |
| 2.31698895 | 12 | 39 | 3 | 49 | 49.5% | 19.7% | 92.9% | 80.0% | 44.3% |
| 2.32931727 | 11 | 39 | 3 | 50 | 48.5% | 18.0% | 92.9% | 78.6% | 43.8% |
| 2.34206471 | 10 | 39 | 3 | 51 | 47.6% | 16.4% | 92.9% | 76.9% | 43.3% |
| 2.40509091 | 9 | 39 | 3 | 52 | 46.6% | 14.8% | 92.9% | 75.0% | 42.9% |
| 2.46341463 | 9 | 40 | 2 | 52 | 47.6% | 14.8% | 95.2% | 81.8% | 43.5% |
| 2.52654545 | 8 | 40 | 2 | 53 | 46.6% | 13.1% | 95.2% | 80.0% | 43.0% |
| 2.52718676 | 7 | 40 | 2 | 54 | 45.6% | 11.5% | 95.2% | 77.8% | 42.6% |
| 2.55264798 | 6 | 40 | 2 | 55 | 44.7% | 9.8% | 95.2% | 75.0% | 42.1% |
| 2.58847737 | 5 | 40 | 2 | 56 | 43.7% | 8.2% | 95.2% | 71.4% | 41.7% |
| 2.6437659 | 4 | 40 | 2 | 57 | 42.7% | 6.6% | 95.2% | 66.7% | 41.2% |
| 2.67032967 | 3 | 40 | 2 | 58 | 41.7% | 4.9% | 95.2% | 0.6 | 0.4081633 |
| 2.93348891 | 2 | 40 | 2 | 59 | 40.8% | 3.3% | 95.2% | 0.5 | 0.4040404 |
| 2.98765432 | 1 | 40 | 2 | 60 | 39.8% | 1.6% | 95.2% | 0.3333333 | 0.4 |
| 2.996139 | 1 | 41 | 1 | 60 | 40.8% | 1.6% | 97.6% | 0.5 | 0.4059406 |
| 3.36710834 | 0 | 41 | 1 | 61 | 39.8% | 0.0% | 97.6% | 0 | 0.4019608 |
| 3.49182764 | 0 | 42 | 0 | 61 | 40.8% | 0.0% | 100.0% | NA | 0.407767 |
